# Extreme Environments Facilitate Hybrid Superiority – The Story of a Successful *Daphnia galeata* × *longispina* Hybrid Clone

**DOI:** 10.1371/journal.pone.0140275

**Published:** 2015-10-08

**Authors:** Johanna Griebel, Sabine Gießler, Monika Poxleitner, Amanda Navas Faria, Mingbo Yin, Justyna Wolinska

**Affiliations:** 1 Department of Ecosystem Research, Leibniz-Institute of Freshwater Ecology and Inland Fisheries, Berlin, Germany; 2 Department of Biology II, Ludwig-Maximilian-University Munich, Planegg-Martinsried, Germany; 3 MOE Key Laboratory for Biodiversity Science and Ecological Engineering, School of Life Science, Fudan University, Shanghai, China; 4 Department of Biology, Chemistry, Pharmacy, Institute of Biology, Freie Universität Berlin, Berlin, Germany; Consiglio Nazionale delle Ricerche (CNR), ITALY

## Abstract

Hybridization within the animal kingdom has long been underestimated. Hybrids have often been considered less fit than their parental species. In the present study, we observed that the *Daphnia* community of a small lake was dominated by a single *D*. *galeata* × *D*. *longispina* hybrid clone, during two consecutive years. Notably, in artificial community set-ups consisting of several clones representing parental species and other hybrids, this hybrid clone took over within about ten generations. Neither the fitness assay conducted under different temperatures, or under crowded and non-crowded environments, nor the carrying capacity test revealed any outstanding life history parameters of this hybrid clone. However, under simulated winter conditions (i.e. low temperature, food and light), the hybrid clone eventually showed a higher survival probability and higher fecundity compared to parental species. Hybrid superiority in cold-adapted traits leading to an advantage of overwintering as parthenogenetic lineages might consequently explain the establishment of successful hybrids in natural communities of the *D*. *longispina* complex. In extreme cases, like the one reported here, a superior hybrid genotype might be the only clone alive after cold winters. Overall, superiority traits, such as enhanced overwintering here, might explain hybrid dominance in nature, especially in extreme and rapidly changing environments. Although any favoured gene complex in cyclic parthenogens could be frozen in successful clones independent of hybridization, we did not find similarly successful clones among parental species. We conclude that the emergence of the observed trait is linked to the production of novel recombined hybrid genotypes.

## Introduction

The role of hybridization in evolutionary and ecological processes in the animal kingdom has long been disregarded. Hybrids are often less fit than their parents, produce less viable offspring or are even sterile (reviewed in [1, 2]). Furthermore, genotypes of the parental species have been evolving under prolonged selective pressure and are therefore highly adapted to their environment. By contrast, recombined hybrid genomes might experience a breakdown of co-adapted gene complexes and, as a result, be less competitive in the same environment (reviewed in [3]). However, in the last decade hybridization came into focus of evolutionary biologists, regarding such processes as speciation [4], heterosis [5] and transgressive hybridization [6]. Moreover, the number of hybrid species discovered among animals is increasing [7–10], showing that the evolutionary significance of hybrids has traditionally been underestimated (e.g. [11]). By combining two different genomes, new genotypes can be created that are pre-adapted to novel and/or extreme habitats [2]. Indeed, hybrids are sometimes observed to have a higher fitness than their parental species [8, 12, 13]. All of these aspects challenge conventional views of the evolutionary outcome of hybridization [4, 14, 15].

In the freshwater cladoceran *Daphnia*, hybridization is common worldwide [12, 16, 17]. Interspecific *Daphnia* hybrids are produced during the sexual reproductive phase of the life cycle of these otherwise cyclical parthenogens. Compared to parental species, hybrids of the *Daphnia longispina* complex (mainly consisting of *D*. *galeata*, *D*. *longispina*, *D*. *cucullata* and their interspecific crosses; taxonomy revised in [18]) often experience lower success in further sexual reproduction. This is because hybrids produce less viable sexual dormant eggs (i.e. which are enclosed in a structure called ephippium) which consequently results in their lower hatching success [19–21]. However, previous field and laboratory surveys have shown that hybrids can compete successfully with their parental species during the parthenogenetic phase of reproduction. Specifically, hybrids were found to display higher fitness values during the parthenogenetic phase of reproduction while being exposed to certain environmental conditions; such as when fed with specific qualities (e.g. [22]) or quantities of algae (e.g. [23]), exposed to specific temperature (e.g. [24]), predation (e.g. [25]) or parasite pressure (e.g. [26]). For example, *D*. *galeata × D*. *longispina* hybrids performed poorly at 20°C but showed higher fitness values when raised at 14°C, compared to their parental species [24]. Environmental changes in time and space may therefore provide open windows for the successful establishment of hybrids in the *Daphnia longispina* complex [27]. In fact, field observations indicate that *Daphnia* hybrids may become more abundant or outcompete their parental species during certain time periods [12, 16, 17]. The diversity of successful traits observed in *Daphnia* hybrids is more likely to be the result of hybridization per se rather than of long-term evolution processes acting on hybrid genomes formed in the past. Based on comparison of the mitochondrial DNA, divergence between hybrids and their parental species is low [28, 29], suggesting recent hybridization events.

Successful *Daphnia* hybrid communities are likely to consist of numerous genotypes, because hundreds of thousands of new genotypes hatch every spring from the sexually produced egg bank (lake sediments often contain large numbers of *Daphnia* diapausing eggs that have been accumulated over dozens of years: 10^3^−10^5^ eggs per square meter [30]). Although the best adapted genotypes will increase their abundance in the community by parthenogenetic reproduction (e.g. [31]), it is unlikely that a single genotype would dominate the entire community. Nevertheless, it has been reported from Africa that a single hybrid genotype of *D*. *pulex* and *D*. *pulicaria* was able to invade and displace an entire lake *Daphnia* community within 60 years after its original introduction from America [32] and is now the unique genotype from the *D*. *pulex* complex present in several lakes in the Rift Valley region [33]. In contrast to the native *D*. *pulex*, this *D*. *pulex* × *D*. *pulicaria* hybrid genotype produces dormant eggs asexually, allowing the reappearance of the hybrid genotype after harsh conditions and, finally, the replacement of local residents. However, thus far, this is the only known evidence of a single *Daphnia* genotype being able to outcompete an entire community.

In a previous study of a small lake in Germany, we observed that a single hybrid genotype (*D*. *galeata* × *D*. *longispina*) dominated the entire community of *Daphnia* [31]. To gain insight into the long-term processes contributing to this success, we screened the abundance of this special hybrid genotype (hereafter referred to as “successful hybrid”) over a six-year period in this follow-up study. Moreover, we tested the competitive advantage of the “successful hybrid” in artificial communities, consisting of parental species and other hybrid genotypes. To define the specific traits which led to the observed dominance of the “successful hybrid” within the natural and artificial communities, we conducted several fitness assays. All these fitness assays focused on the parthenogenetic phase of the *Daphnia* reproductive cycle. Specifically, we compared the life history parameters of the “successful hybrid” versus other genotypes of the parental species and hybrids (hereafter referred to as “clones”), under two different temperatures and under crowded- and non-crowded conditions. The applied experimental conditions were based on previous findings. Thus, certain temperature regimes can enhance the performance of *D*. *galeata × D*. *longispina* hybrids when compared to their parental species [24]. Additionally, crowded conditions can induce alteration in life history parameters among species of the *D*. *longispina* complex [34]. We also compared the carrying capacity of the “successful hybrid” with other clones, expecting the “successful hybrid” to exhibit the highest values for this trait. Indeed, carrying capacity can differ between clones of *Daphnia* [35] as it may be linked with the efficient use of limited food resources. Lastly, life history traits were compared under simulated winter conditions. This was done because the “successful hybrid” was detected across several years of the field survey, and must therefore have survived winter as a parthenogenetic lineage.

## Material and Methods

### Ethics statement

Collection of zooplankton (*Daphnia*) in this study did not require specific permissions, and our study did not involve the use or collection of endangered or protected species.

### Abundance of the “successful hybrid”

Feldmochinger See, Munich (48°12’50”N, 11°30’50”E), is a small (0.16 km^2^) and shallow (max. depth of 5 m) artificial lake (flooded gravel pit), created in 1939. The lake is mesotrophic (OECD guidelines) with about 30 mg/m^3^ total phosphorus, 3220 mg/m^3^ nitrogen and 1.8 mg/m^3^ chlorophyll a (average values of March to November 2009, data provided by Andreas Scholz and Marion Duschl, Wasserwirtschaftsamt München: www.wwa-m.bayern.de). We traced the development of the “successful hybrid” in this lake by analysing zooplankton samples collected in the spring of six years (April to early June, until the first occurrence of *Daphnia* individuals, see also S1 Fig), between 2008 and 2013 (data from the 2008–2011 period have been published elsewhere as part of a geographical survey of *Daphnia* communities from several lakes [31, 36]). Additional zooplankton samples were collected from 2011–2013, monthly throughout the growing season (from April until October/November). Zooplankton samples were collected at two sites within the deep basin of the lake (depth was measured using a portable depth sounder), by hauling a plankton net (95 μm mesh size) through the whole water column. The two samples were pooled and preserved in 96% ethanol. Then, if available, about 50–90 adult females from the *D*. *longispina* complex were randomly selected per sample for genotyping. For the 2011–2013 collections, a random subsample was used for *Daphnia* density estimates (only adult females were considered).

*Daphnia* were genotyped at 15 microsatellite loci [37] in two multiplex polymerase chain reactions (MP1: Dgm105, Dgm112, SwiD5, SwiD7, SwiD8; and MP2: Dgm109, Dp196, Dp281, Dp512, SwiD1, SwiD2, SwiD10, SwiD12, SwiD14, SwiD15), following the protocol described in Yin, Wolinska [36]. Genotypes were scored using GeneMapper version 3.7 (Applied Biosystem). Short size differences between runs were adjusted by using a locus-specific pattern of a certain reference clone that was included in each run. GenALEx 6.5 [38] was applied for the identification of multilocus genotypes (MLGs). Relying on the 2011 genotype data, where the hypothesis that identical MLGs were of sexual origin was rejected (GENECLONE 2.0: p < 0.001) [31], individuals with same MLGs were assumed to represent members of the same clone. Based on their MLG, *Daphnia* individuals were assigned by NewHybrids 1.1 [39] into six possible classes (two parental species and four hybrid classes: F1, F2 and both backcrosses). Additionally, 35 well-defined reference clones of *D*. *galeata*, *D*. *longispina* and their hybrids (for a list of clones see [36]) were included in the NewHybrids analysis to verify species assignment.

Finally, abundance patterns of the “successful hybrid” were compared to the strength of winters using the i) North Atlantic Oscillation Index (NAO-index, data from https://climatedataguide.ucar.edu/climate-data/hurrell-north-atlantic-oscillation-nao-index-station-based), ii) the average air temperature of Munich in corresponding winters (data from www.wetterkontor.de) and iii) the maximum thickness of ice during winter (lakes are sometimes frozen, except the region of effluent at the north, data provided by Siegfried Lechner from the Baureferat Gartenbau Munich). The NAO-Index describes the difference between the high subtropical surface pressure in the Azore High and the low surface pressure in the Icelandic Low [40]. A large difference in pressure between the two locations leads to increased westerlies, resulting in warm winters, while in the opposite case westerlies are decreased and winters are cold. Therefore a positive/negative index value is correlated with a warm/cold winter and higher/lower water temperatures in European lakes [41, 42]. Specifically, Pearsons’s correlation coefficient was calculated between the strength of the winter (i.e. NAO-index, air temperature or thickness of ice) and the abundance of the “successful hybrid”.

### Experimental *Daphnia* clones

The “successful hybrid” was isolated from Feldmochinger See in May 2011 and kept in the laboratory as a clonal lineage from then on. To evaluate the competitive performance of the “successful hybrid” relative to other *Daphnia* genotypes, several clones were isolated from nine other small flooded gravel pits (in and around Munich), and from one natural pre-alpine lake close to Munich, Ammersee (a list of sampling sites is provided in Table 1, while their geographical locations can be seen in Fig 2 in Yin, Gießler [31]). Sampling sites were separated by 50 km at most. Established clonal lineages were genotyped at 15 microsatellite loci. Individuals were then assigned by NewHybrids into one of the six possible classes (see above). From this clonal collection, 16 *D*. *galeata*, 15 *D*. *longispina* and 7 F1-hybrid clones (all representing different MLGs) were then randomly selected for further experimental tests (for a list of clones and their origin see Table 1). Additionally, two clones of another cladoceran species, *Simocephalus* (family *Daphnidae*), were isolated from zooplankton samples, to be used later in the competition experiment (to introduce interspecific competition).

**Table 1:** List of clones used in the specific experiments (indicated by ×): clonal ID, origin, sampling date, taxon membership (based on 15 microsatellite loci and NewHybrid assignment).

clonal ID	origin	sampling date	taxon	competition	temperature	crowded	carrying capacity	overwintering
AMME_10	Ammersee	29.09.2008	*D*. *galeata*	**×**	**×**			
AMME_24	Ammersee	29.09.2008	*D*. *galeata*	**×**	**×**			
AMME_47	Ammersee	11.11.2008	*D*. *galeata*	**×**	**×**		**×**	**×**
AMME_58	Ammersee	18.12.2008	*D*. *galeata*	**×**				
AMME_66	Ammersee	18.12.2008	*D*. *galeata*	**×**				
FASA_01	Fasanerie See	20.04.2011	*D*. *galeata*	**×**	**×**	**×**	**×**	**×**
FASA_07	Fasanerie See	20.04.2011	*D*. *galeata*	**×**	**×**		**×**	
FASA_13	Fasanerie See	20.04.2011	*D*. *galeata*	**×**	**×**			
HEIM_05	Heimstettner See	18.05.2011	*D*. *galeata*	**×**				
HEIM_06	Heimstettner See	18.05.2011	*D*. *galeata*	**×**		**×**	**×**	**×** ^†^
HEIM_08	Heimstettner See	18.05.2011	*D*. *galeata*	**×**	**×**			
HEIM_12	Heimstettner See	18.05.2011	*D*. *galeata*	**×**				
HEIM_14	Heimstettner See	18.05.2011	*D*. *galeata*	**×**	**×**		**×**	**×**
LERC_09	Lerchenauer See	20.04.2011	*D*. *galeata*	**×**	**×**			
LERC_11	Lerchenauer See	20.04.2011	*D*. *galeata*	**×**	**×**			
LERC_33	Lerchenauer See	20.04.2011	*D*. *galeata*	**×**	**×**			
AMME_38	Ammersee	29.09.2008	*D*. *longispina*	**×**	**×**	**×**	**×**	**×** ^†^
LANG_08	Langwieder See	19.04.2011	*D*. *longispina*	**×**	**×**		**×**	**×**
LANG_21	Langwieder See	19.04.2011	*D*. *longispina*	**×**	**×**			
LANG_26	Langwieder See	19.04.2011	*D*. *longispina*	**×**	**×**			
LUSS_12	Lußsee	19.04.2011	*D*. *longispina*	**×**	**×**			
LUSS_30	Lußsee	18.05.2011	*D*. *longispina*	**×**	**×**			
OLCH_02	Olchinger See	18.04.2011	*D*. *longispina*	**×**	**×**			
OLCH_17	Olchinger See	18.04.2011	*D*. *longispina*	**×**	**×**			
OLCH_22	Olchinger See	18.04.2011	*D*. *longispina*	**×**				
OLCH_29	Olchinger See	18.04.2011	*D*. *longispina*	**×**	**×**	**×**	**×**	**×** ^†^
WALD_03	Waldschwaigsee	18.04.2011	*D*. *longispina*	**×**	**×**			
WALD_05	Waldschwaigsee	18.04.2011	*D*. *longispina*	**×**	**×**			
WALD_12	Waldschwaigsee	18.04.2011	*D*. *longispina*	**×**				
WALD_16	Waldschwaigsee	18.04.2011	*D*. *longispina*	**×**			**×**	**×** ^†^
WALD_37	Waldschwaigsee	18.04.2011	*D*. *longispina*	**×**				
AMME_03	Ammersee	18.09.2008	F1-hybrid	**×**	**×**			
AMME_12	Ammersee	29.09.2008	F1-hybrid	**×**		**×**	**×**	**×**
AMME_61	Ammersee	18.12.2008	F1-hybrid	**×**	**×**		**×**	**×**
“successful hybrid”	Feldmochinger See	18.05.2011	F1-hybrid	**×**	**×**	**×**	**×**	**×**
FERI_01	Feringasee	18.04.2011	F1-hybrid	**×**	**×**			
FERI_14	Feringasee	18.04.2011	F1-hybrid	**×**	**×**		**×**	**×** ^†^
LUSS_04	Lußsee	19.04.2011	F1-hybrid	**×**	**×**		**×**	**×**
BOHM_01	Böhmer Weiher	19.05.2011	*Simocephalus*	**×**				
BOHM_03	Böhmer Weiher	19.05.2011	*Simocephalus*	**×**				

^†^ Individuals of a given clone failed to reproduce during the acclimation period and the respective clone was therefore excluded from the final experiment.

### Genetic relatedness of the “successful hybrid”

First, it has been tested if hybrid MLGs represent distinct clones, instead of multilocus lineages (MLL) (resulting from scoring errors or somatic mutations [43]) Thus, the frequency of pairwise distances (number of distinct alleles per locus) between all hybrid genotypes of the Feldmochinger See was calculated using GenClone 2.0 [44]. Then, as a control distribution, hybrid genotypes were simulated by random combinations of genotypes from the parental species (by using R software). For the simulation, a *D*. *galeata* genotype was randomly chosen from the 2009 Feldmochinger See sample, while a *D*. *longispina* genotype was randomly selected from a nearby lake (Waldschwaigsee), sampled in 2009. The shape of data distributions was compared between detected and simulated hybrid genotypes; a bimodal distribution with an initial peak at low differences would indicate the presence of scoring errors or somatic mutations [45]. Furthermore, to evaluate genetic relatedness among hybrid genotypes, MLGs of the F1-hybrids collected from Feldmochinger See and from three other lakes (i.e. all F1-hybrid clones used in the experiments) and two reference F1-hybrids [36] were used to compute Nei’s pairwise genetic distances [46] for UPGMA (Unweighted Pair-Group Method with the Arithmetic Mean) clustering analysis. Analyses were performed in PHYLIP 3.69 [47] with 10^3^ bootstrap replications. Genetic relatedness of the experimental genotypes was displayed by applying a factorial correspondence analysis (FCA) on the MLGs in Genetix 4.0 [48].

### Experimental conditions

Prior to all experiments, clones were raised under standard experimental conditions for at least two generations, to reduce maternal effects. Unless varied in a certain experiment (see below), these conditions included: 20 ± 0.5°C (walk-in chamber), artificial SSS media [49], 12:12 light-dark photoperiod, and a daily diet of 1 mg C L^-1^ *Scenedesmus obliquus*, a laboratory grown green algae. *S*. *obliquus* were raised in Z-media [50], using 20 *±* 0.5°C and a 20:4 light-dark photoperiod. For an overview of the specific experimental conditions for each experiment, see S1 Table.

### Competition experiment

To measure the competitive strength of the “successful hybrid”, five different experimental assemblages were set up from 38 available *Daphnia* clones and two *Simocephalus* clones. Each experimental assemblage consisted of a different combination of twelve clones (but always included four clones of *D*. *galeata*, four clones of *D*. *longispina*, two F1-hybrid clones, the “successful hybrid” and one *Simocephalus* clone: S2 Table). Prior to the experiment, about 150 individuals from each clone were kept separately in jars containing 1 L of medium (two to four such jars were set up per clone and six jars for the “successful hybrid”). Then, 17 individuals per clone were placed together (except clones OLCH_17 and FERI_14, for which only twelve individuals were available) to form five clonal sets of about 200 *Daphnia* per jar. The starting proportion was thus 8%–9% for each clone. Per clonal set, two conditions (treatments) were tested, with four replicates each (resulting in 40 experimental units). In the first treatment (high initial density), all individuals of the twelve represented clones were placed together in 4 L glass jars, containing 1 L of medium. Medium was then added to each jar at a rate of 200 mL every two days, until a volume of 4 L was reached. In the second treatment (low initial density), all *Daphnia* were initially placed in jars containing a 4 L volume of medium. In both treatments, *Daphnia* were fed every third day with 1.0 mg C L^-1^ *S*. *obliquus* and the positions of the jars were randomized. The following parameters were recorded: 1) population density and 2) final relative frequency of genotypes. After ten weeks, *Daphnia* individuals were counted (adults) and 24 adult females were randomly selected per jar for genotyping. Here, ten microsatellite markers were used (MP2, see above), as this was sufficient to identify the experimental clones. Identification of the clonal origin of each individual (based on the clone-specific MLG) was performed in GenALEx 6.5 [38]. The proportion of individuals from the “successful hybrid” and the final densities of the adults were analysed using a two-way ANOVA with the starting density (two levels: high and low) and the different sets of clones (five levels: clonal set 1–5) as factors. As the proportion of individuals from the “successful hybrid” was not normally distributed, values were arcsine-transformed prior to the analysis to allow the application of a parametric test. Concerning the density of *Daphnia* individuals, three outliers were detected, according to the QQ-Plot (data not shown). After removing these values, the data were normally distributed (the results of the ANOVA did not change if the outliers were included in the analysis). Further pairwise comparisons were performed using Tukey’s HSD. All statistical tests (here and elsewhere) were performed in R 3.0.2 [51].

### Temperature experiment

Life history characteristics of the “successful hybrid” were compared with 28 other clones (Table 1), exposed to two different temperature treatments. Prior to the experiment, approximately ten *Daphnia* individuals from a single clone were kept in 200 mL of medium (four to five jars per clone), at a temperature of 18 ± 0.5°C. The newborns (<24h) from the second and third clutch were randomly distributed to jars with 40 mL media and subjected to the two temperature treatments: 15°C and 20°C (temperature was reduced/raised stepwise over three days). First, two to three *Daphnia* individuals were placed in each jar (six to ten replicates per clone and treatment, depending on the extent of the observed male production at 18°C) because high mortality was expected in the first phase of the experiment, due to experimental handling. On day five, however, only one of those individuals was randomly selected to continue the experiment. Half of the media was refreshed every third day and the positions of the jars were randomized at the same time. The following parameters were recorded: 1) age at first clutch release, 2) number of offspring in the first clutch, 3) total number of offspring in the first three clutches, 4) first clutch offspring body length (5 offspring were measured and an average value was taken for statistical tests), 5) body length of experimental mothers at the end of the experiment. The offspring were removed and counted daily. The experiment was terminated for each *Daphnia* individually, on the day after the release of the third clutch. Individuals that did not reproduce were checked for gender and males were excluded from further analyses. The body length measurements (of frozen-in-water individuals) were taken with a digital image-analysis system (Cell^P, Olympus, Hamburg, Germany), from the upper end of the eye to the base of the posterior spine. Per parameter, differences among the clones were tested using the Kruskal-Wallis test; separately per temperature treatment (none of the collected parameters were normally distributed). Then, pairwise comparisons between the “successful hybrid” and each of the other clones were conducted. Additionally, differences between performance at 20°C and 15°C were tested per clone, using the Mann-Whitney U-test. *P*-values were adjusted for multiple comparisons with Bonferroni-Holm correction [52]. Most individuals of six clones (Table 1) died before reproducing at 15°C and these clones were then excluded from the Kruskal-Wallis (15°C) and Mann-Whitney U-tests. Furthermore, to visualize summarized life history differences between the “successful hybrid” and the other clones, a principal component analysis (PCA) based on the five parameters measured was applied at the individual level. Individuals with missing values were excluded from the PCA (i.e. 98 out of 327 cases).

### Crowded conditions experiment

Life history characteristics of the “successful hybrid” were compared with five other clones (Table 1) under two different conditions (crowded and non-crowded media). Prior to the experiment, about ten *Daphnia* from each clone were kept separately in 200 mL medium (four to five jars per clone). The offspring (<24h) from the second clutch were randomly distributed between the two media treatments in 40 mL volume. Ten replicates were set up per clone and treatment. First, two to three *Daphnia* individuals were placed in each jar (again, because high mortality was expected in the first phase of the experiment due to experimental handling). On day five, however, only one of those individuals was randomly selected to continue the experiment. Crowded medium was obtained from batch cultures containing a mixture of *Daphnia* clones representing both parental species and hybrids, kept in a 4 L volume (four jars). Densities of *Daphnia* individuals in crowded cultures varied between 200 and 500 individuals/L. The same method of producing “crowded medium” was used in previous experimental studies [34, 53]. The “successful hybrid” was not present in these cultures to ensure a mixture of genotypes in the media. Similarly, 4 L jars were set up for non-crowded media (i.e. without *Daphnia*). After 48 hours, 3 L of crowded (and non-crowded) media were filtered (mesh size: 0.45 μm) and used to replace half of the media in the experimental jars. This partial medium replacement was done daily and, at the same time, the positions of the jars were randomized. The same procedure was applied as described in the temperature experiment to retrieve and analyse life history data. Individuals with missing data were excluded from the PCA (i.e. 55 of 120 cases).

### Carrying capacity experiment

In this experiment the carrying capacity of the “successful hybrid” was compared with 13 other clones (Table 1). Prior to the experiment, about 150 *Daphnia* individuals from each clone were kept in 1 L medium (2–4 jars clone). Then, ten adult *Daphnia* of a given clone were placed in 200 mL of medium (6 replicates per clone). *Daphnia* were fed with 1.0 mg C L^-1^ *S*. *obliquus* and the position of the jars was randomized every third day. Half of the media in experimental jars was refreshed weekly and all *Daphnia* were counted (i.e. regardless of age and gender) by pipetting them to a new jar. The experiment was terminated after seven weeks. For each week the per capita growth rate (change in numbers of *Daphnia* per count) was calculated and plotted against population size. The carrying capacity was obtained for each replicate using linear regression (assuming logistic growth, [54]), by calculating the intercept point with the x-axis [55, 56]. A one-way ANOVA was applied with clone (14 levels) as a factor. Further, pairwise comparisons of the carrying capacity of the clones were performed using Tukey’s HSD.

### Overwintering experiment

Here, life history parameters of the “successful hybrid” were compared with twelve other clones, while simulating winter conditions. The same twelve clones were used as in the carrying capacity experiment (Table 1). Prior to the experiment, approximately ten *Daphnia* from each clone were kept in 200 mL medium (four to five jars per clone). The temperature was reduced stepwise. Thus, two generations before the start of the experiment, individuals were raised at 16°C, while their offspring (generation -1) were raised at 12°C. Experimental offspring (generation 0) of eight clones (the other clones failed to reproduce) were then randomly distributed to jars with 40 mL of medium (15 replicates per clone) and raised, for two days, at 8°C. Experimental conditions were: 4±0.5°C, 8:16 light-dark photoperiod and a diet of 0.1 mg C L^-1^ *S*. *obliquus* applied every second day. Half of the media was refreshed weekly and the positions of the jars were randomized. The following parameters were recorded: 1) lifespan, 2) total number of offspring from all clutches, 3) total number of clutches, 4) number of ephippia produced. The offspring were removed and counted every second day. The experiment was terminated when the last experimental individual died. Survival probability through time was calculated for each of the clones by using Cox Proportional Hazard Models [57]. The base model was computed using the data of the “successful hybrid”. The total number of offspring from all clutches, the total number of clutches as well as the number of ephippia produced were added as covariates in the model. The best fitted model was tested using Schoenfelds residuals [58]. Statistical test were performed using the survival packages in R [59].

## Results

### Abundance of the “successful hybrid”

**Fig 1:**
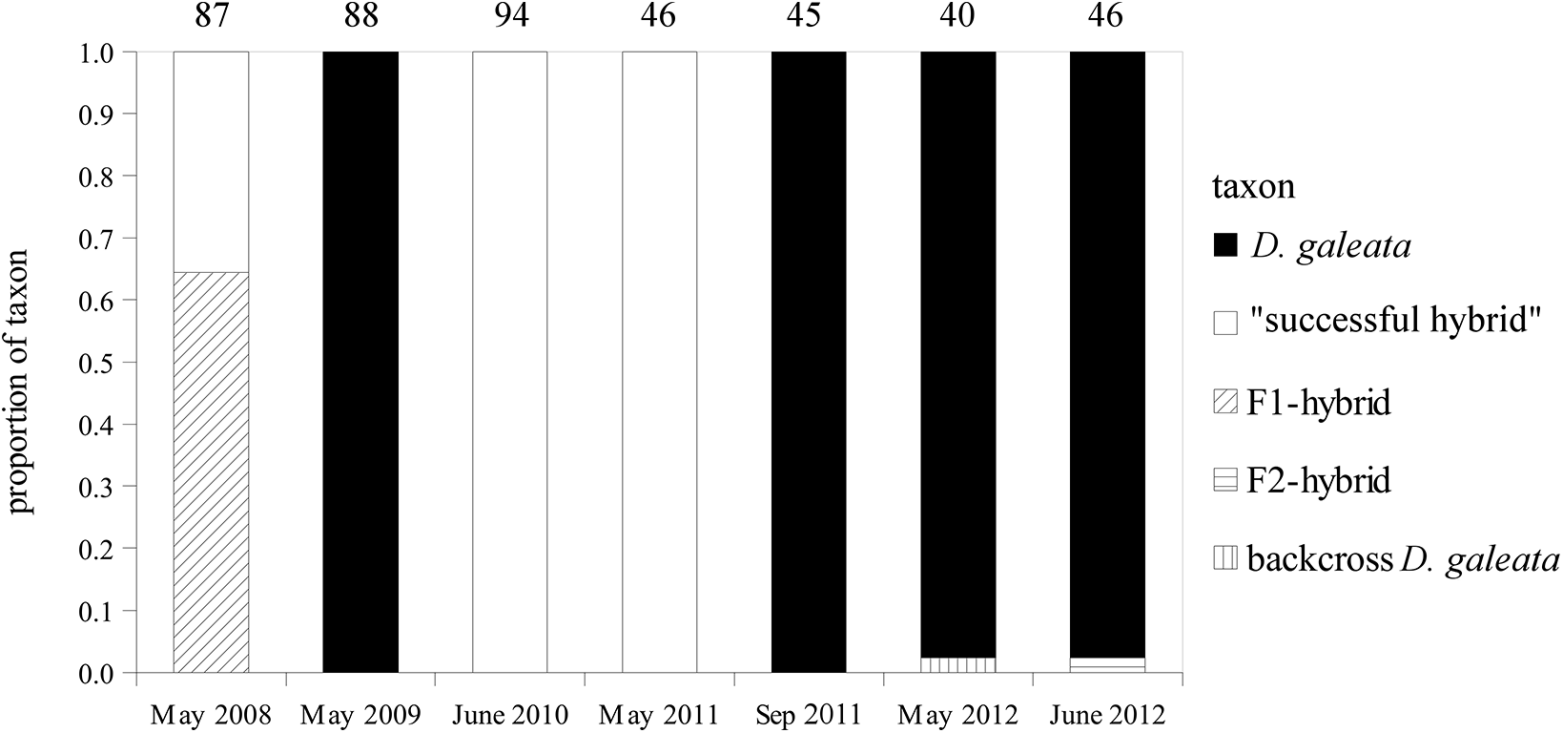
Daphnia taxon composition in the Feldmochinger See between 2008 and 2012, based on 15 microsatellite loci and NewHybrids assignment. The “successful hybrid” dominated the entire community in spring 2010 and 2011. The numbers above the bars show the number of genotyped individuals.

*Daphnia* were detected in the Feldmochinger See in spring samples of all studied years (2008–2013) and in four additional samples from the monthly collections performed during the years 2011–2013 (S1 Fig). Densities of *Daphnia* were often low and, altogether, only seven samples contained enough individuals for genotyping, resulting in a total of 481 genotyped individuals. Based on NewHybrids assignments, mainly *D*. *galeata* and F1-hybrids (*D*. *galeata × D*. *longispina*) were present in the studied lake (Fig 1). In spring 2008, the “successful hybrid” reached a proportion of 36% (it co-existed with other F1-hybrids at that time). In spring 2009, however, its abundance was below detection level (only *D*. *galeata* was detected in that sample). In spring of 2010 and 2011, all genotyped *Daphnia* represented the “successful hybrid” (94 and 46 individuals, respectively). In autumn 2011, the “successful hybrid” was again replaced by *D*. *galeata*, which remained dominant in 2012 (Fig 1). In 2013, not enough *Daphnia* were found for genotyping. Comparison of the NAO-Index with the abundance patterns of the “successful hybrid” revealed that, in winter 2009/2010, after which the “successful hybrid” was the only abundant clone, the NAO-Index was -2.54 (the lowest value since the start of the record in 1870). In winter 2010/2011 the NAO-Index was still negative (-0.91) and the “successful hybrid” was again the only abundant clone. For winter 2011/2012, the NAO-Index was highly positive (2.08) and the “successful hybrid” did not appear again (S2 Fig). Additionally, the average winter temperature was below zero in winters before the years when the “successful hybrid” was the only abundant clone, compared to most other winters. Nevertheless, in the winter with the second most negative NAO-Index (i.e. 2010/2011; after which the “successful hybrid” was the only abundant clone), no ice was detected on the Feldmochinger See. Overall, the association between the strength of the winter and the relative abundance of the “successful hybrid” was neither significant for the NAO-Index nor for the average winter temperature or the thickness of ice (S2 Fig).

### Genetic relatedness of the “successful hybrid”

**Fig 2:**
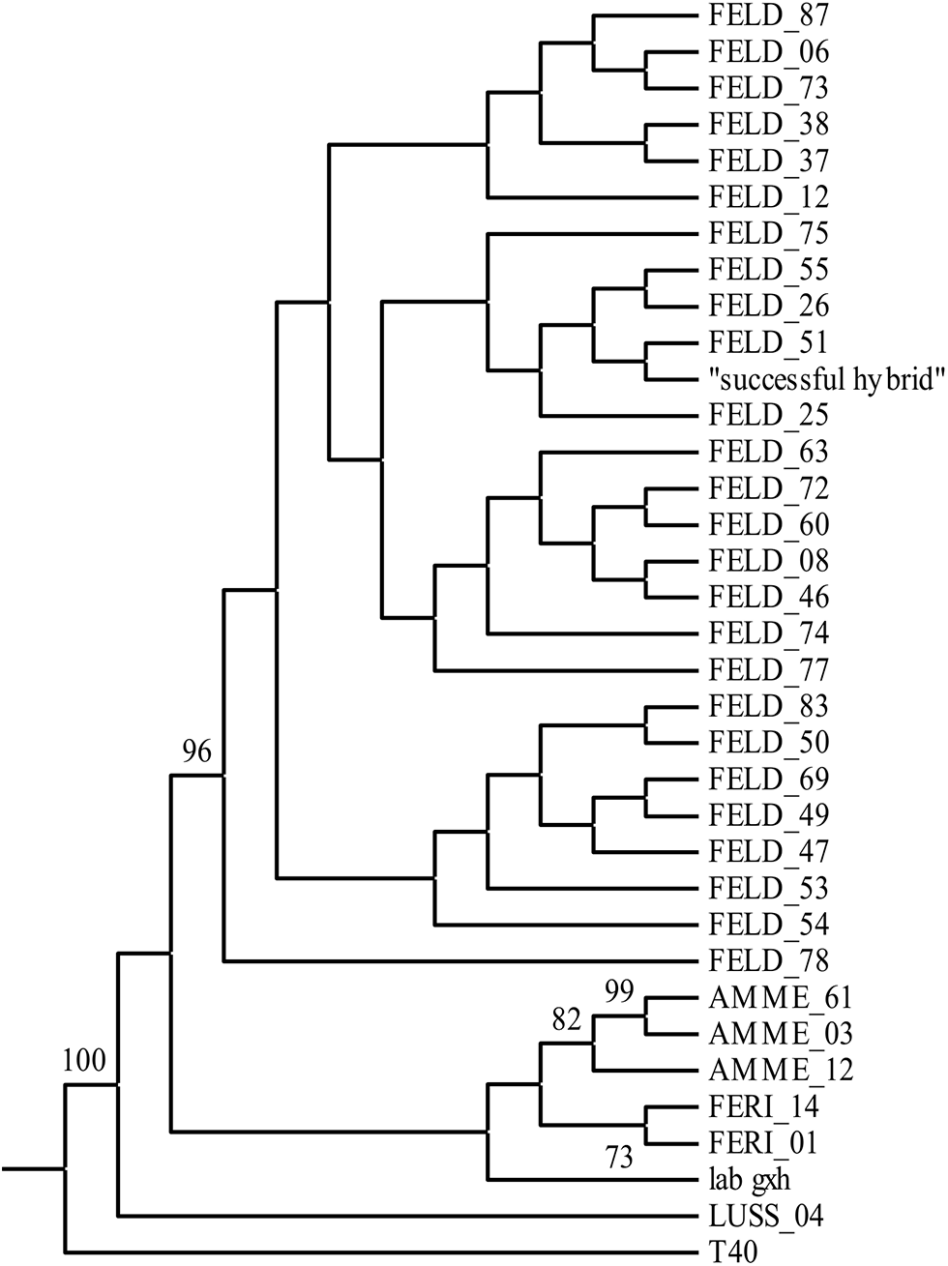
UPGMA clustering of Nei’s genetic distance based on the microsatellite data of the F1- hybrid individuals sampled from the Feldmochinger See. In addition, F1-hybrids used in the experiments (originating from three different lakes: Ammersee, Lußsee, Feringasee; see Table 1) and two reference F1-hybrids (T40 and lab g×h [29]) are included in the tree. The “successful hybrid” clusters with other F1-hybrids from the Feldmochinger See. Only bootstrap values > 50% are shown.

The frequency distribution of pairwise distances (number of distinct alleles) among all MLG showed an unimodal distribution with no extra peak at low distances (S3 Fig), indicating that all MLGs represent different clones. Genotypic similarities among clones (based on microsatellite data) showed that the “successful hybrid” clustered with other F1-hybrids from the Feldmochinger See on the UPGMA tree (Fig 2). Additionally, the intermediate hybrid position of the “successful hybrid” compared to other F1-hybrid experimental clones can be seen on the FCA plot based on the allelic variation at the 15 microsatellite loci (S4 Fig).

### Competition experiment

**Fig 3:**
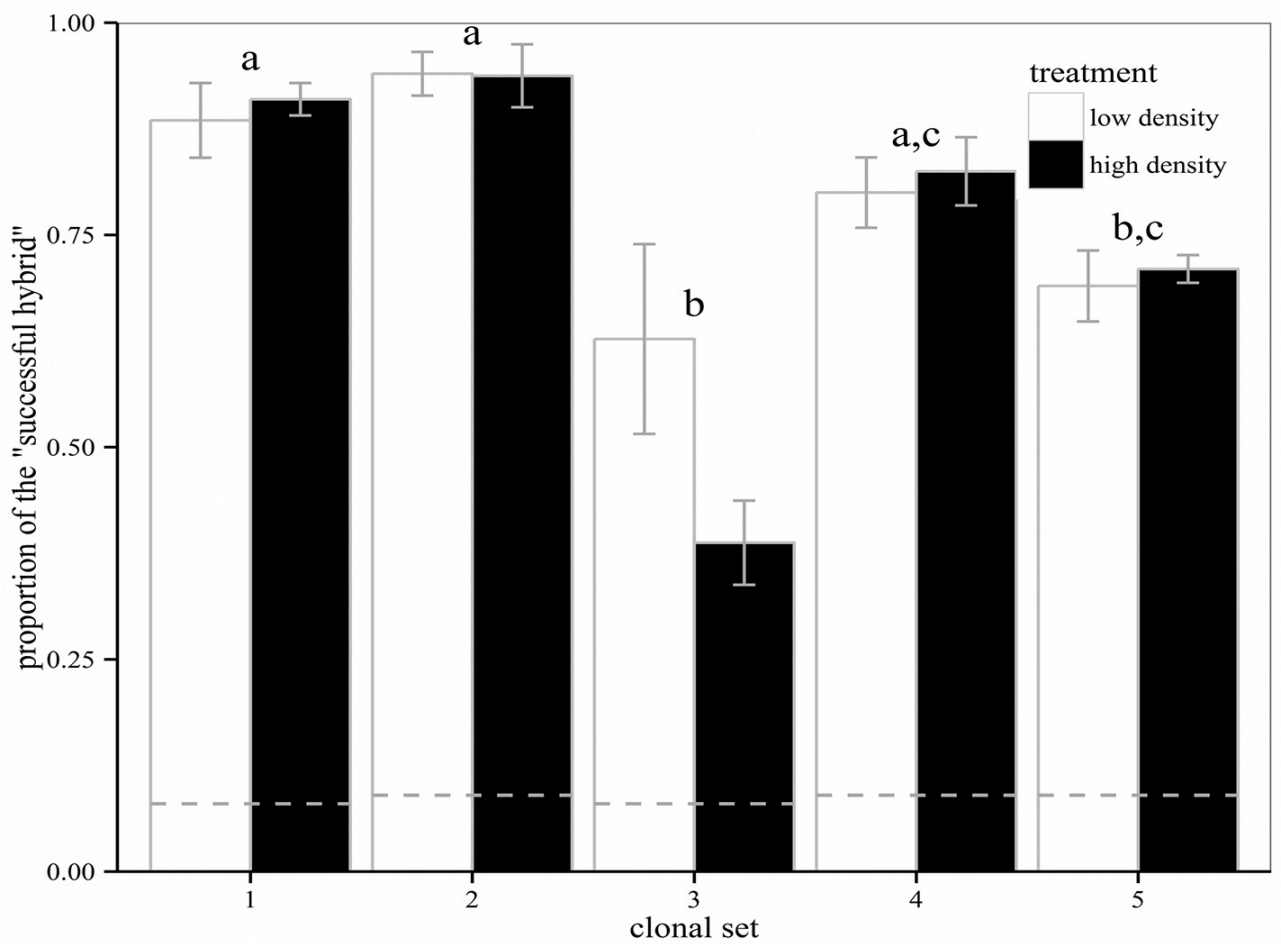
Competition experiment. Comparison of the final proportion of the “successful hybrid” among clonal sets (1–5) in two density treatments. Shown are the means ± SE. The dashed lines specify the starting proportions of the “successful hybrid”. The “successful hybrid” increased its abundance across all clonal sets and both density treatments. However, its final density differed across clonal sets (same letters above the columns indicate no significant differences between the respective clonal sets).

The proportion of the “successful hybrid” substantially increased within a period of ten weeks from the original 8–9%: the highest proportion was reached in clonal set no. 2 (94% and 93%, in the high and low density treatment, respectively), and the lowest proportion in clonal set no. 3 (62% and 38%, respectively: Fig 3; F_4,36_ = 20.6, *p*<0.001). In clonal set no. 3, the two most abundant clones besides the “successful hybrid” were also F1-hybrids. Neither the starting density (F_1,36_ = 0.5, *p* = 0.46) nor the clonal set × starting density interaction (F_4,36_ = 1.4, *p* = 0.25) had a significant effect on the proportion of the “successful hybrid”. The final proportion of *Simocephalus* was low in all treatments (0.0–0.3%). The final densities of experimental populations varied between 99 and 188 *Daphnia*/L (i.e. averaged per clonal set and treatment). The clonal set (F_4,36_ = 3.3, *p* = 0.02) as well as the starting density × clonal set interaction (F_4,36_ = 3.4, *p* = 0.02), but not starting density alone (F_1,36_ = 0.2, *p* = 0.632), had a significant effect on final densities (S5 Fig).

### Temperature experiment

The performance of the “successful hybrid” was mainly intermediate for the measured parameters, only sometimes showing values significantly higher or lower than those of any other clone (Table 2). Indeed, multivariate analysis using PCA visualized intermediate life histories for individuals of the “successful hybrid” in the two-dimensional axis-plot (S6 Fig). Regarding differences between temperatures, most of the clones released the first clutch earlier at 20°C than at 15°C (S3 Table). Also, some clones reached a greater body length and/or produced larger offspring at 20°C than at 15°C.

**Table 2:** Results of the temperature, crowded conditions and carrying capacity experiments. H^2^ or F-values, degrees of freedom (d.f.) and p-values are shown for each dependent variable and treatment. In addition, parameter values significantly different between the “successful hybrid” and other clones (after Bonferroni-Holm corrections) are indicated with </> (i.e. “successful hybrid” had a smaller or larger value, respectively).

experiment	dependant variable	treatment	d.f.	H^2^/F	*p*	“successful hybrid” vs. others clones
temperature	age at 1^st^ clutch release	20°C	28	80.44	<0.001	<LANG_21, <WALD_16
		15°C	22	65.54	<0.001	
	no. of offspring in the 1^st^ clutch	20°C	28	50.76	0.005	
		15°C	22	39.54	0.012	
	total no. of offspring in the 1^st^ three clutches	20°C	28	80.51	<0.001	>LUSS_30
		15°C	22	51.65	0.006	
	1^st^ clutch offspring body length	20°C	28	54.45	0.002	>FASA_01, >LANG_26
		15°C	22	40.12	0.01	
	body length experimental mothers	20°C	28	94.57	<0.001	>AMME_61, >AMME_38, >LANG_26, >WALD_03
		15°C	22	88.96	<0.001	<LERC_33, >LANG_26, >WALD_03
crowded conditions	age at 1^st^ clutch release	crowded	5	24.65	<0.001	<AMME_12, <AMME_38
		non-crowded	5	8.27	0.14	
	no. of offspring in the 1^st^ clutch	crowded	5	21.34	<0.001	<OLCH_29
		non-crowded	5	13.93	0.01	<OLCH_29, >HEIM_06
	total no. of offspring in the 1^st^ three clutches	crowded	5	23.98	<0.001	<OLCH_29, >AMME_12
		non-crowded	5	18.64	0.002	<OLCH_29
	1^st^ clutch offspring body length	crowded	5	18.96	0.002	>FASA_01
		non-crowded	5	21.64	<0.001	>AMME_12
	body length experimental mothers	crowded	5	27.43	<0.001	<OLCH_29, <HEIM_06, >AMME_38,
		non-crowded	5	16.65	0.005	>FASA_01
carrying capacity	carrying capacity	-	13	21.24	<0.001	>FERI_14, >FASA_07, <HEIM_14,<OLCH_29, <FASA_01

### Crowded conditions experiment

The performance of the “successful hybrid” was mainly intermediate for the measured parameters, only sometimes showing values significantly higher or lower than those of any other clone (Table 2). Again, multivariate analysis using PCA displayed intermediate life histories for the “successful hybrid” (S7 Fig). Regarding the response to different media types, all clones showed a greater body length in crowded compared to non-crowded media. Three clones had a higher total number of offspring and larger 1^st^ clutch offspring size in crowded media than in non-crowded media (S3 Table). These differences between the media treatments can also be seen on the PCA-plot (S7 Fig). The “successful hybrid” was the only clone that released its first clutch earlier in non-crowded than in crowded media (S3 Table).

### Carrying capacity experiment

The measured carrying capacity varied between clones, from 65 to 280 individuals per litre (F_13,68_ = 21.28, *p*<0.001). However, the carrying capacity of the “successful hybrid” did not show any outstanding values (Table 2, S8 Fig).

### Overwintering experiment

**Fig 4:**
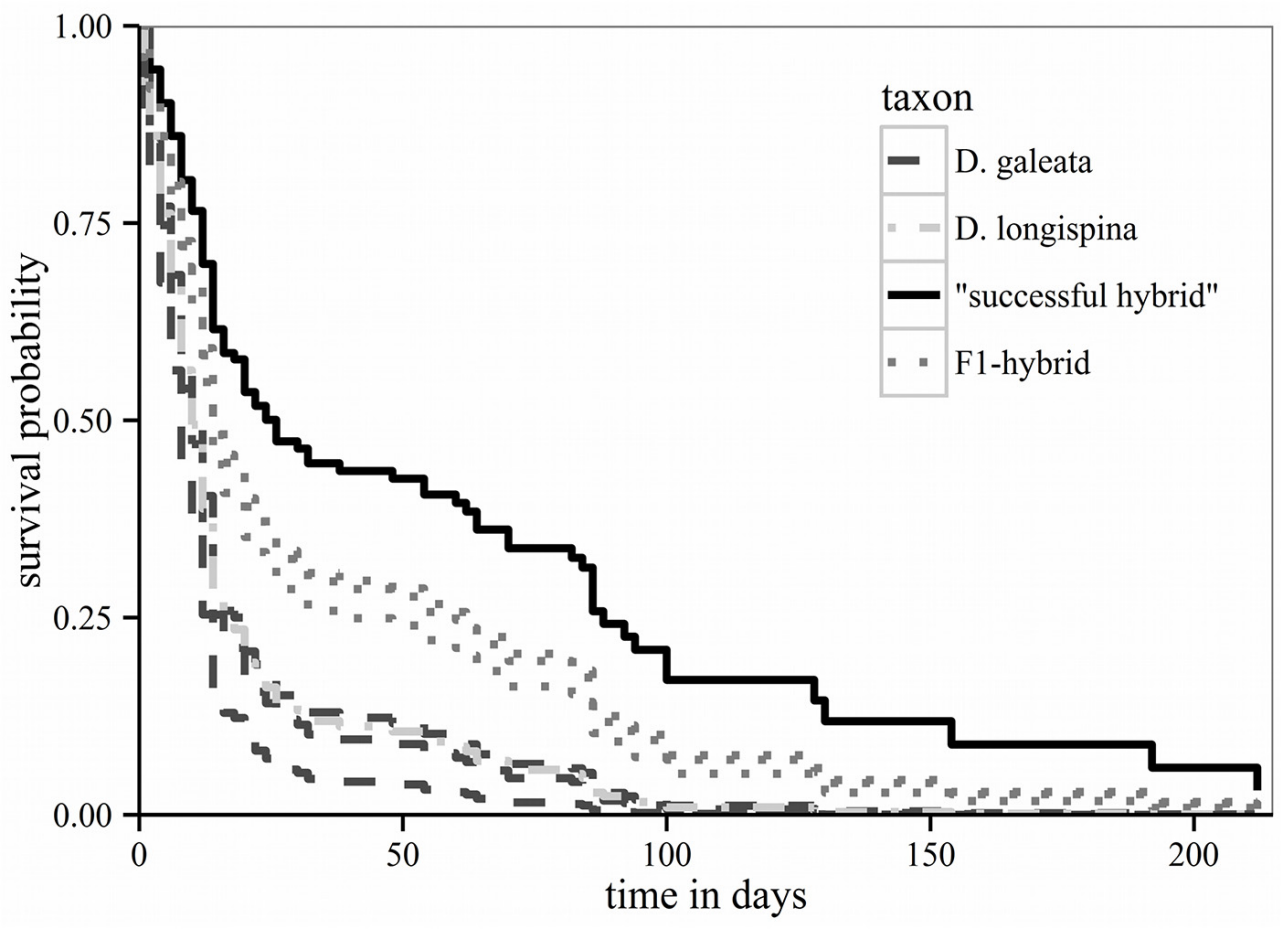
Winter experiment. Predicted survival curves estimated in the Cox regression model for each clone over time. The “successful hybrid” is represented by a solid line. Other hybrid clones are represented by dotted lines, species clones are indicated by dashed lines. The “successful hybrid” shows the highest survival probability followed by F1-hybrids and parental species.

The experiment was set up with eight out of the thirteen clones that were initially taken for the acclimation period (Table 1), due to the failure of the other five clones to reproduce at 16°C or at 12°C. The best fitted Cox Proportional Hazards Model was obtained with the total number of clutches and the total number of offspring included as covariates (S9 Fig). Both covariates showed a decreasing effect on the hazard ratios (Table 3). The hazard ratios of all *D*. *galeata* and *D*. *longispina* clones were three times higher than the hazard ratio of the “successful hybrid” (Table 3), whereas the other hybrids showed no significant difference on the hazard ratios compared with the “successful hybrid”. The survival curve of the “successful hybrid” was higher than that of other clones (the maximum observed lifespan was 212 days). Additionally, the survival curves of other hybrid clones were intermediate compared with the “successful hybrid” and clones of parental species (Fig 4). Per clone, at least one individual reproduced; in total, 421 new-borns were released (only females). In the case of the “successful hybrid”, three of 15 tested experimental individuals reproduced; each releasing more than four clutches and a large number of offspring (S10 Fig). Only one other clone (hybrid AMME_61) produced more than four clutches. In total, only three individuals produced ephippia (each of them belonging to a different clone: “successful hybrid”, LUSS_04 or LANG_08).

**Table 3:** Winter experiment. Results of the Cox-regression model, HR: hazard ratio, 95%-CI: confidence interval of the hazard ratio, z-value (Wald statistics) and p-value. The “successful hybrid” represents the base line of the model.

clonal ID	taxon	HR	95%-CI	z	*p*
“successful hybrid”	F1-hybrid				
AMME_47	*D*. *galeata*	3.97	1.8–8.5	3.51	<0.001
FASA_01	*D*. *galeata*	3.70	1.7–8.0	3.39	<0.001
HEIM_14	*D*. *galeata*	3.08	1.4–6.6	2.88	0.003
LANG_08	*D*. *longispina*	2.16	1.0–4.6	1.99	0.04
AMME_12	F1-hybrid	1.24	0.5–2.6	0.56	0.57
AMME_61	F1-hybrid	2.00	0.9–4.4	1.70	0.08
LUSS_04	F1-hybrid	1.55	0.7–3.2	1.14	0.25
total number of clutches		0.66	0.5–0.8	-2.89	0.003
fecundity		0.93	0.8–1.0	-2.46	0.01

## Discussion

The outcome of competition between parthenogenetic lineages of hybrids and their parental species is expected to be determined by genotype-by-environment interactions [1]. The genomes of parental species might be well adapted to their local environments, whereas hybrids might experience a breakdown of co-adapted gene complexes (reviewed in [3]). However, in novel or frequently changing habitats the creation of new and unique genotypes via hybridization allows for rapid adaptation to otherwise sub-optimal conditions [2]. Small shallow lakes are highly variable environments, often affected by seasonal changes such as cold temperatures during winter. In such a variable habitat studied here, a single *Daphnia* hybrid genotype outcompeted all other *Daphnia* genotypes, during two consecutive years. Remarkably, in addition to being superior to clones from the same lake under natural conditions, the “successful hybrid” was the most abundant clone in artificial communities, where, within ten weeks, it outcompeted *Daphnia* clones originating from several other lakes. The success of this clone as observed in artificial communities (run under 20°C and using two different initial population densities), however, cannot be explained by the performance of this clone in any of the life-history surveys conducted across different ecological settings. This is because the performance of the “successful hybrid” was similar to other clones; at both tested temperatures (20°C and 15°C), in crowded and non-crowded media, and with respect to carrying capacity. Different outcomes of life history surveys and competition experiments have been reported previously in *Daphnia* systems [60, 61]. Thus, fitness parameters responsible for success under competition might have been overlooked in simplified life history assays in the laboratory, or the measured traits might change when facing competition [62]. Because life history assays will never perfectly mimic the natural field conditions, it is unlikely to identify additional superior traits of the “successful hybrid” in the laboratory. For example, planktivorous fish, including carp, are highly abundant in Feldmochinger See (data from local fishing association AFG), which might explain the low *Daphnia* densities in the lake (S1 Fig). Thus, a potential adaptive reaction of the “successful hybrid” to predation pressure (see e.g. [63, 64]) could additionally contribute to its temporal advantage.

Importantly, no media exchange was performed in the competition experiment conducted here compared to the life history assays. Keeping such dense cultures of *Daphnia* in the same media for ten weeks might have resulted in unusually high bacteria growth due to decomposed corpses of *Daphnia* or algal cells. It has been shown across many hybridizing complexes, that hybrids express novel traits allowing survival in extreme habitats [65–68]. For example, in spadefoot toads hybrids displayed lower fitness under normal conditions compared to the parental species, but shorter developmental time, enabling them to escape death if ponds dry out early in the season [66]. Another examples are sunflowers, where production of extreme hybrid phenotypes enabled the colonization of sand dunes, desert floor and salt marsh habitats [68].

The “successful hybrid” (and other hybrid clones) studied here had higher survival probabilities compared to the parental species, when exposed to simulated winter conditions. To overcome winter, *Daphnia* can either produce sexual dormant eggs that are deposited in the sediment and hatch in the following spring, or survive in the water column as parthenogenetic (i.e. asexual) females [69]. The strategy of overwintering as an asexual female could become advantageous in spring: surviving females can immediately reproduce parthenogenetically, and their offspring can profit from lack of competitors, and from being born during an algal bloom [70]. In contrast, individuals hatching from sexual dormant eggs first need to grow and mature before they are able to reproduce [71]. However, overwintering as an asexual female incurs the risk of death due to winter declines in temperature and food resources [72]. *Daphnia* can display a mixed strategy, first reproducing sexually and then trying to survive winter as a clonal lineage [69]. In general, hybrids are known to be less successful in sexual reproduction than their parental species [19]. Therefore, temporal hybrid advantage is linked to the parthenogenetic part of the life cycle and the likelihood of hybrids contributing to the next spring population would indeed be increased by having a higher probability of surviving winter as asexual females.

The observed here enhanced overwintering of the “successful hybrid” as asexual female can either be the result of hybrid superiority per se, or might result from of a special adaptation in this clone independent from its hybrid origin. In cyclical parthenogens favourite gene complexes of the parents may be frozen within their asexually reproducing descendants according to the frozen niche model (FNV, [73, 74]), as genotypes are not broken up due to sexual reproduction. Favoured traits of these descendant genotypes can be associated with special niches at the edges of the parental environments leading to coexistence or even dominance. Independent of inter- or intraspecific origin, such clones are assumed to be successful in other habitats sharing similar environmental settings [73]. However, neither the “successful hybrid” was detected in any of the nearby lakes [31] nor any “frozen” parental genotype (i.e. showing increased overwintering or other successful characteristics) was detected across the experimental surveys, even though also genotypes of the parental species are in principle able to persist over several years [75]. In fact, the “successful hybrid” was similar to any other clone in most of the tested life history parameters. The latter observation would then be consistent with the general-purpose-genotype model (GPG, [73, 74]) postulating a broad adaptation of a clone across many life history traits. However, the fecundity and survival probability during winter conditions of the “successful hybrid” and other hybrid clones were higher compared to the parental species, suggesting additive genetic effects caused by hybridization as a source of their superiority. In consequence, the occurrence of the “successful hybrid” seems to be the result of a combination of different evolutionary processes.

The success of a novel hybrid trait, such as enhanced overwintering as asexual females detected here, might depend on environmental conditions (e.g. [69]). The Feldmochinger See is a small and shallow habitat. As those habitats often experience rapid declines in water temperature, *Daphnia* living there would benefit from being adapted to harsh winters, facilitating the occurrence of genotypes with enhanced overwintering abilities, such as the “successful hybrid”. The fact that the “successful hybrid” was closely grouped to other hybrids sampled from the Feldmochinger See community (based on UPGMA-clustering), provides evidence that this clone was produced by local parental species rather than being a migrant (similarly, it is unlikely that all these hybrid genotypes were migrants, as they would need to originate from the same foreign population then). Indeed, in a similar overwintering experiment as conducted in our study, *Daphnia* clones from the same species complex showed a much shorter lifespan (up to 125 days) and a lower number of offspring produced per fecund female (1–5) [72], compared to up to 212 days and 2–81 offspring per fecund female in our experiment. In contrast to *Daphnia* from Feldmochinger See, however, the other clones originated from large and deep pre-alpine lakes, which might be less drastically affected by winter. Thus, these other *Daphnia* [72] might not have been exposed to selective pressure strong enough to favor certain genotypes possessing these adaptive traits. It has to be noted, however, that the second highest fecundity in the overwintering experiment was observed for the hybrid clone that also originated from a large and deep pre-alpine lake Ammersee (AMME-61). Additional parameters than high fluctuations in hydrodynamic factors (correlates to the small size and low depth in the lake Feldmochinger See) might be selecting for overwintering success in hybrids.

The potentially superior trait of the “successful hybrid”, i.e. overwintering strategy through asexual reproduction, is further supported by the fact that this clone was the only one (among all hybrid and parental lineages as sampled from Feldmochinger See) that was detected across more than one year. Thus, its clonal offspring must have survived winter in the water column, while other clones might have failed at that stage. Indeed, the survival and later dominance of the “successful hybrid” seems to be triggered by strong winters. Even though no significant correlation between strength of winter and the abundance of the “successful hybrid” was detected with the available data (i.e. low statistical power of a test with six data points only); the “successful hybrid” dominated the *Daphnia* communities after two winters with lowest NAO indices. Surprisingly, in the winter with the second most negative NAO-Index (i.e. 2010/2011; after which the “successful hybrid” was the only abundant clone), no ice was detected on the Feldmochinger See. Thus, in this system, the thickness of ice was not correlated with the average winter temperature within the study period of six years. In general, the observed, here only week, association between the strength of the winter (measured as NAO-Index or the average winter temperature) and the relative abundance of the “successful hybrid” (S2 Fig) might be explained by inter-annual variation in several additional parameters. For example, the quantity and quality of food might be a more significant determinant for the niche of a given clone, and not the duration of the ice cover but primary light penetration determines the composition of phytoplankton assemblages [76]. Moreover, the success of this clone in the competition experiment performed at warmer temperatures indicates other possible advantageous traits of the “successful hybrid” that have not been revealed yet. Notably, the consistently observed success of this hybrid clone both in natural conditions and when competing with other clones in a laboratory survey, supports the view that the combination of new traits expressed in hybrid genotypes is of evolutionary significance. The link between recombined preadapted parental gene complexes and clonal reproduction might facilitate the dominance of successful hybrids in extreme or rapidly changing environments.

## Supporting Information

S1 Fig Density of *Daphnia* (individuals/L) in Feldmochinger See from April 2011 until October 2013. Missing values represent months where no sampling was conducted. For 2008–2010 period no density data were collected.
(PDF)

S2 Fig Proportion of the “successful hybrid” in the first spring sample of the Feldmochinger See from 2008 to 2012, in relation to the North Atlantic Oscillation (NAO)-Index, the average winter air temperature (T in°C) and the maximum thickness of ice coverage. r: Spearmans’s correlation coefficient, p: *p*-value.
(PDF)

S3 Fig Distribution of the frequency of the pairwise number of allele differences between MLGs from the hybrids of the Feldmochinger See and simulated hybrid genotypes. (PDF)

S4 Fig Factor loadings derived from factorial correspondence analysis (FCA) based on allelic variation at 15 microsatellite loci. FCA scores of the first two axes are shown for the experimental clones. Parental species (*D*. *galeata*, *D*. *longispina*) and F1-hybrids clusters are encircled. The “successful hybrid” is shown with a filled symbol.
(PDF)

S5 Fig Competition experiment. Comparison of the density of adult *Daphnia* among clonal sets (1–5) and between two starting densities. The error bars show standard error. Same letters above the columns indicate no significant differences between the respective clonal sets.
(PDF)

S6 Fig Temperature experiment. Summary of life history data from clones used in the temperature experiment. PCA analysis based on five parameters: P1: age at 1^st^ clutch release; P2: number of offspring in the 1^st^ clutch; P3: total number of offspring in the first three clutches; P4: body length of experimental mothers, P5: 1^st^ clutch offspring body length. Filled symbols show the individuals of the “successful hybrid”, other clones are displayed by empty symbols. Results from different temperature treatments are indicated by triangles (15°C) and circles (20°C).
(PDF)

S7 Fig Summary of life history data from clones used in the crowded conditions experiment. PCA analysis based on five parameters: P1: age at 1^st^ clutch release; P2: number of offspring in the 1^st^ clutch; P3: total number of offspring in the first three clutches; P4: body length of experimental mothers, P5: 1^st^ clutch offspring body length. Filled symbols show the individuals of the “successful hybrid”, other clones are displayed by empty symbols. Results from different temperature treatments are indicated by circles (non-crowded) and triangles (crowded).
(PDF)

S8 Fig Comparison of carrying capacities among clones. For taxon memberships see Table 1. Shown are the means ± S.E. Same letters above the columns indicate no significant differences between the respective clones.
(PDF)

S9 Fig Scaled Schoenfeld residuals for the eight clones used in the overwintering experiment. In addition, number of clutches and fecundity versus time is shown for the Cox proportional hazard model fit. The solid lines (βt) give the estimated effect of the predictors through time in the experiment (with 95% confidence interval).
(PDF)

S10 Fig Total number of offspring in the overwintering experiment. Values are provided per each individual replicate, but only for those individuals that have reproduced at least once. For taxon membership see Table 1.
(PDF)

S1 Table Overview of the conditions of each experiment. (PDF)

S2 Table Assignment of the clones to the different clonal sets in the competition experiment. (PDF)

S3 Table Differences in life history parameters between two temperatures (15°C vs 20°C) and crowded conditions (C: crowded vs NC: non-crowded), tested separately per clone. The direction of a difference is indicated by < / > signs. Significant *p-*values after Bonferroni-Holm correction are shown in bold.
(PDF)
