# Supplementary figures and images for: Extreme Environments Facilitate Hybrid Superiority – The Story of a Successful Daphnia galeata × longispina Hybrid Clone
Source: PLoS One. 2015 Oct 8;10(10):e0140275. doi: 10.1371/journal.pone.0140275 (PMC4598010; doi:10.1371/journal.pone.0140275)

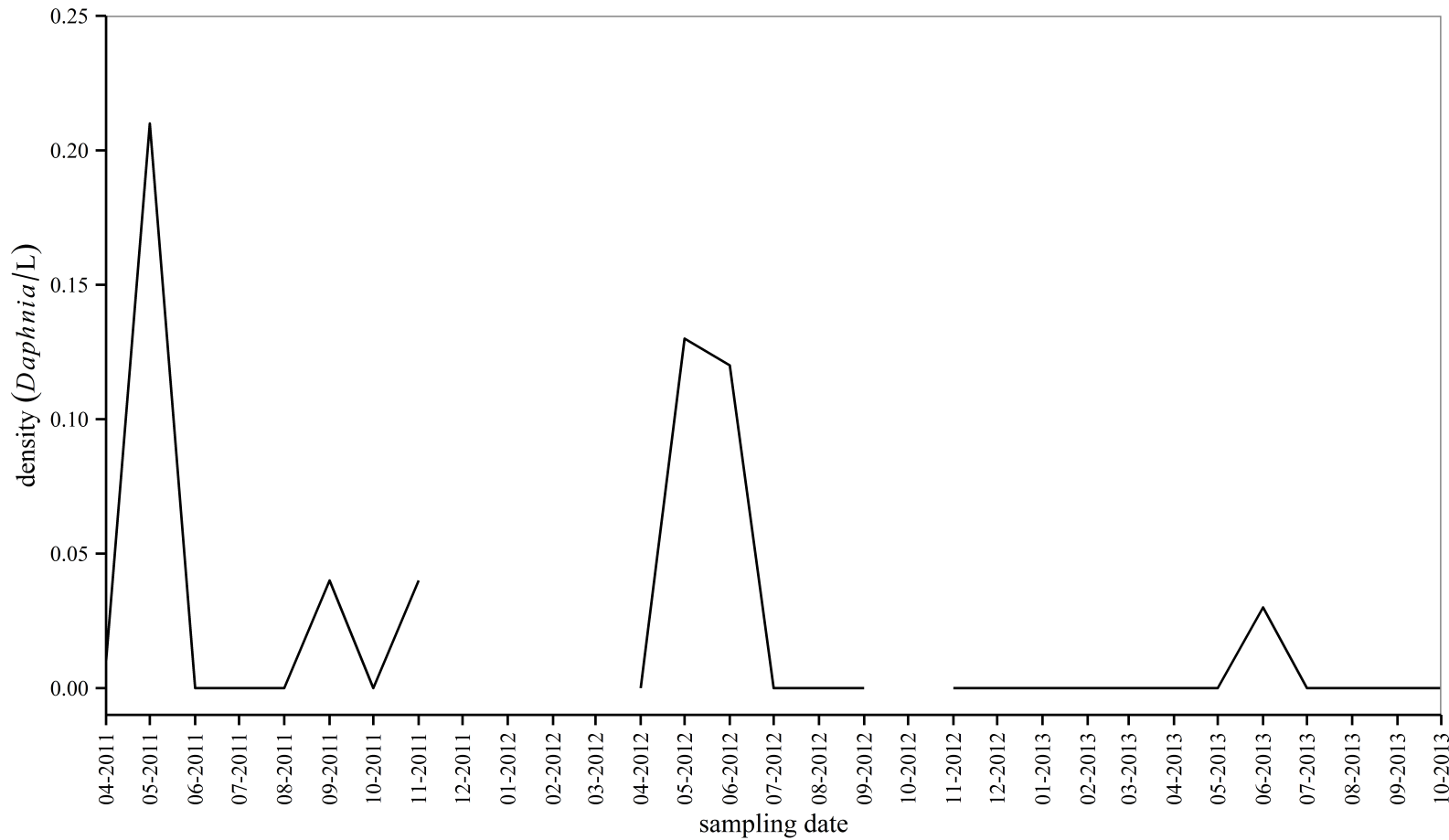

Supplement: S1 Fig — Missing values represent months where no sampling was conducted. For 2008–2010 period no density data were collected. (PDF) [file pone.0140275.s001.pdf]

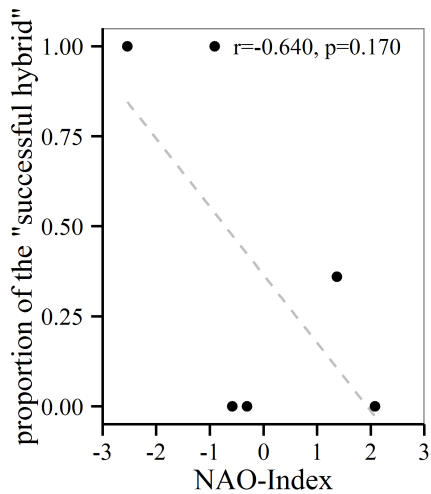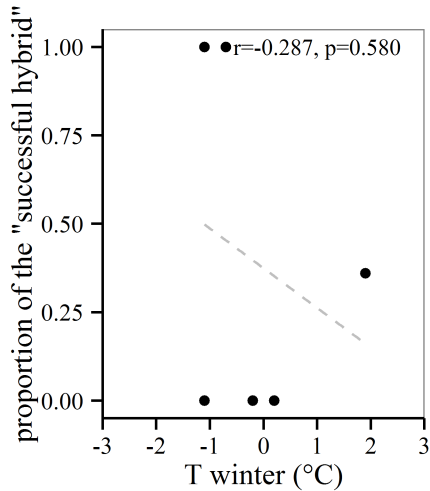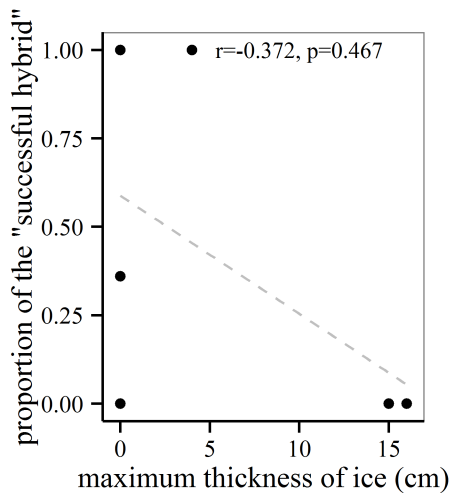

Supplement: S2 Fig — r: Spearmans’s correlation coefficient, p: p-value. (PDF) [file pone.0140275.s002.pdf]

simulated hybrids

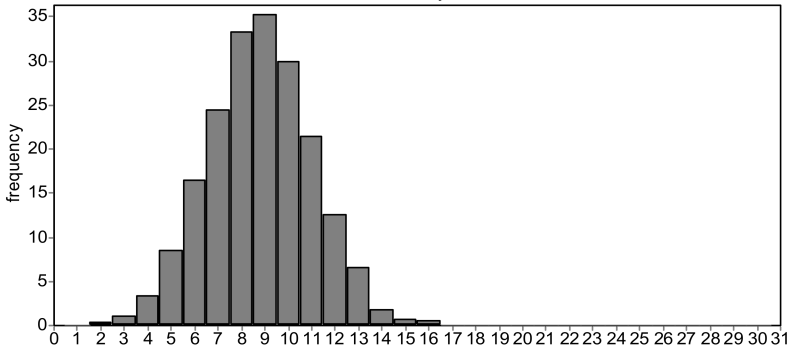

hybrids of the Feldmochinger Lake

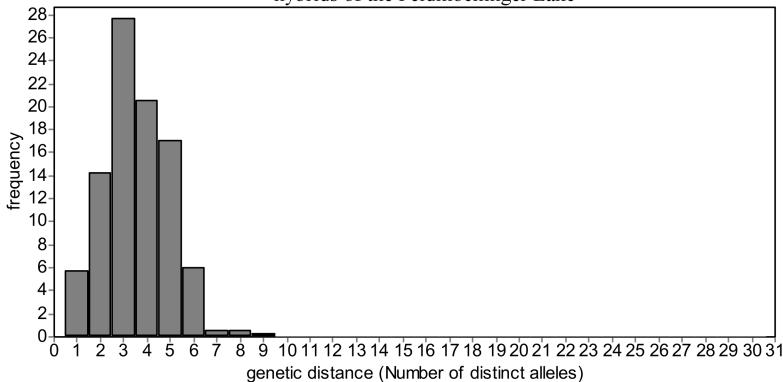

Supplement: S3 Fig — (PDF) [file pone.0140275.s003.pdf]

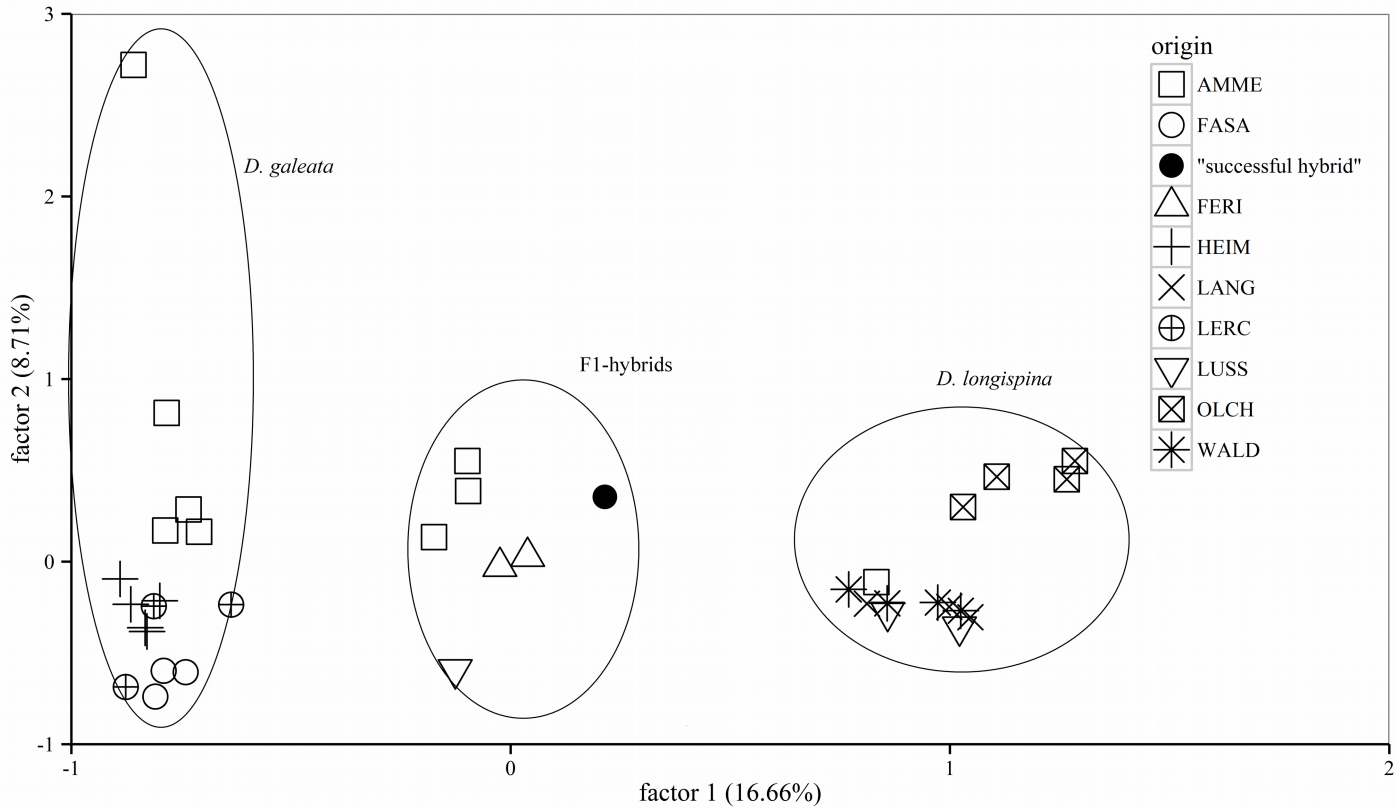

Supplement: S4 Fig — FCA scores of the first two axes are shown for the experimental clones. Parental species (D. galeata, D. longispina) and F1-hybrids clusters are encircled. The “successful hybrid” is shown with a filled symbol. (PDF) [file pone.0140275.s004.pdf]

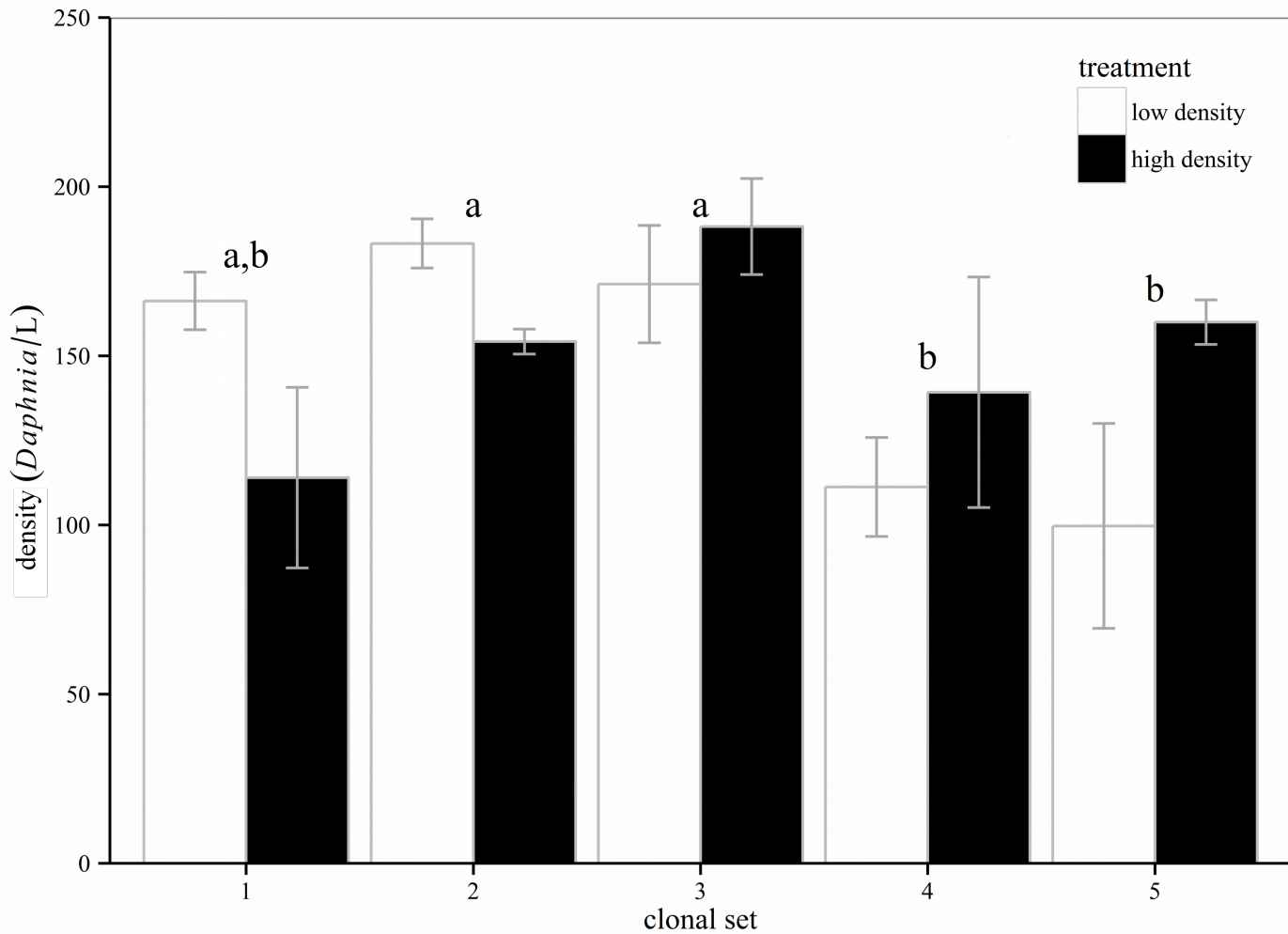

Supplement: S5 Fig — Comparison of the density of adult Daphnia among clonal sets (1–5) and between two starting densities. The error bars show standard error. Same letters above the columns indicate no significant differences between the respective clonal sets. (PDF) [file pone.0140275.s005.pdf]

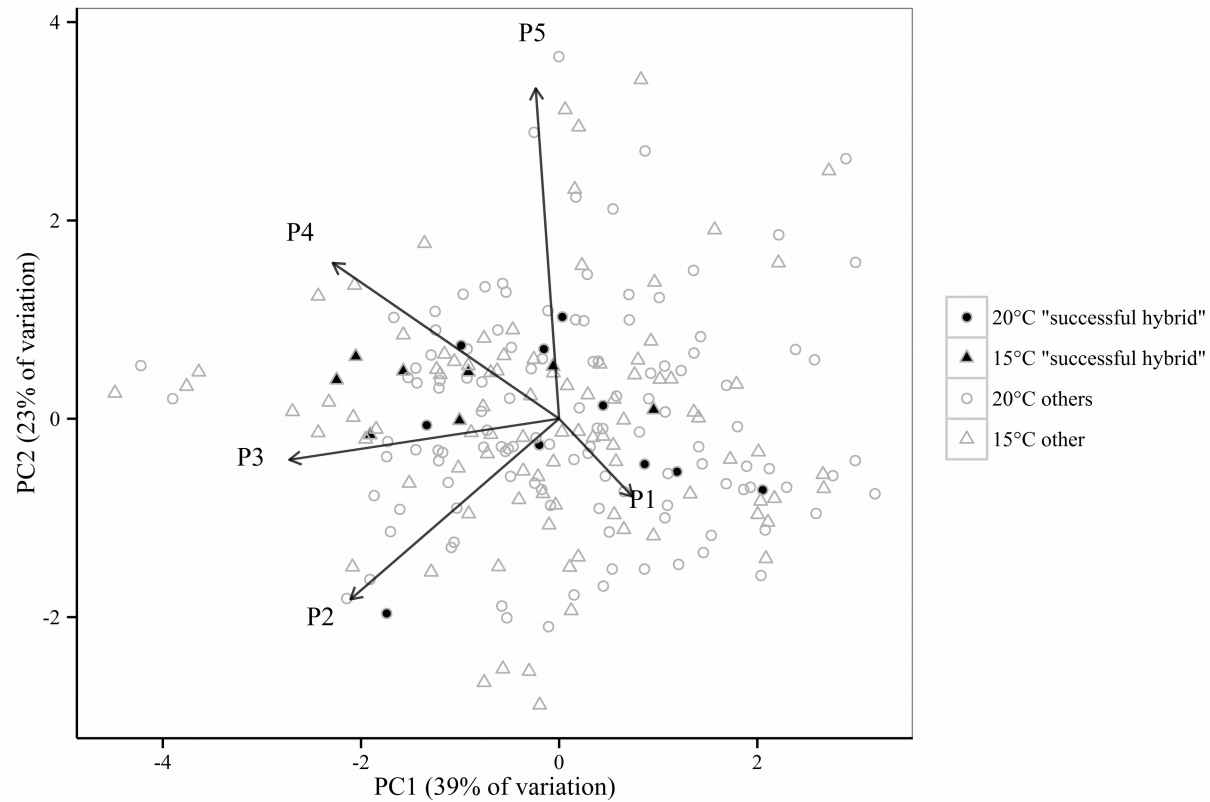

Supplement: S6 Fig — Summary of life history data from clones used in the temperature experiment. PCA analysis based on five parameters: P1: age at 1st clutch release; P2: number of offspring in the 1st clutch; P3: total number of offspring in the first three clutches; P4: body length of experimental mothers, P5: 1st clutch offspring body length. Filled symbols show the individuals of the “successful hybrid”, other clones are displayed by empty symbols. Results from different temperature treatments are indicated by triangles (15°C) and circles (20°C). (PDF) [file pone.0140275.s006.pdf]

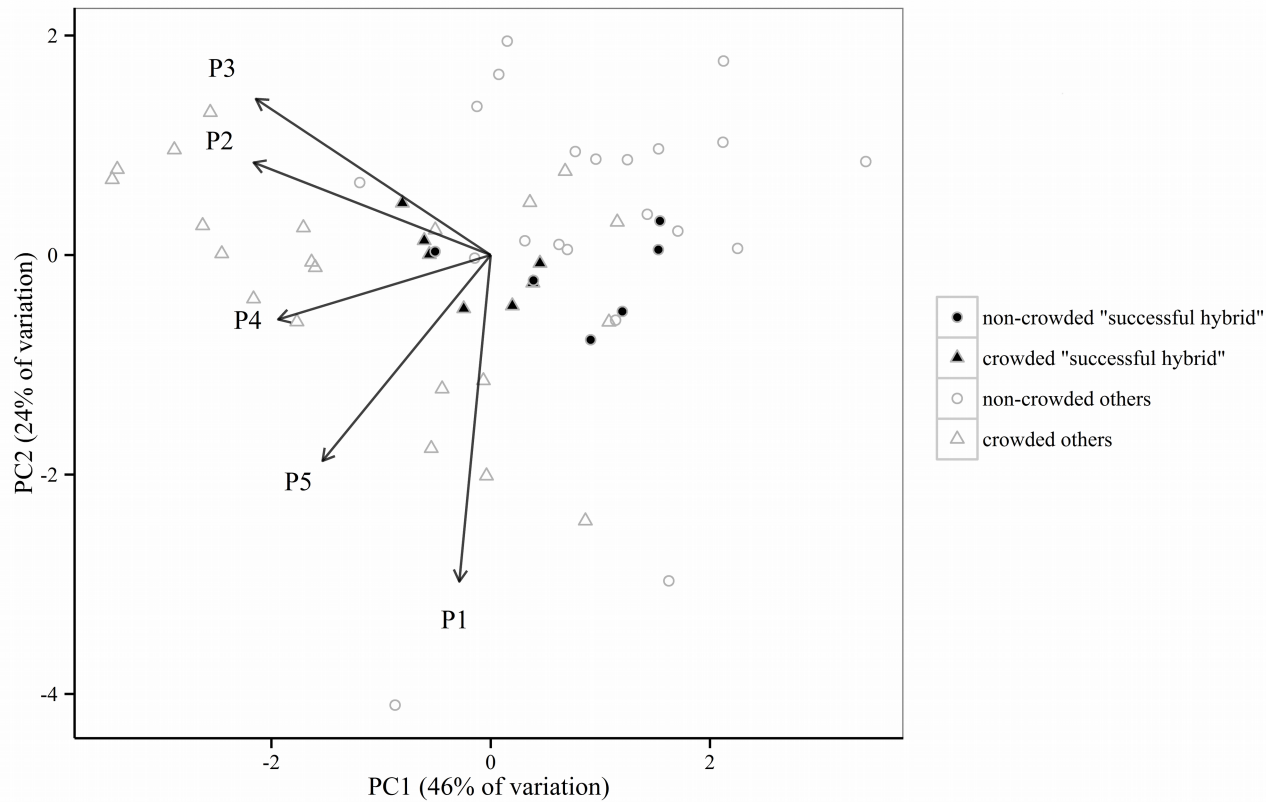

Supplement: S7 Fig — PCA analysis based on five parameters: P1: age at 1st clutch release; P2: number of offspring in the 1st clutch; P3: total number of offspring in the first three clutches; P4: body length of experimental mothers, P5: 1st clutch offspring body length. Filled symbols show the individuals of the “successful hybrid”, other clones are displayed by empty symbols. Results from different temperature treatments are indicated by circles (non-crowded) and triangles (crowded). (PDF) [file pone.0140275.s007.pdf]

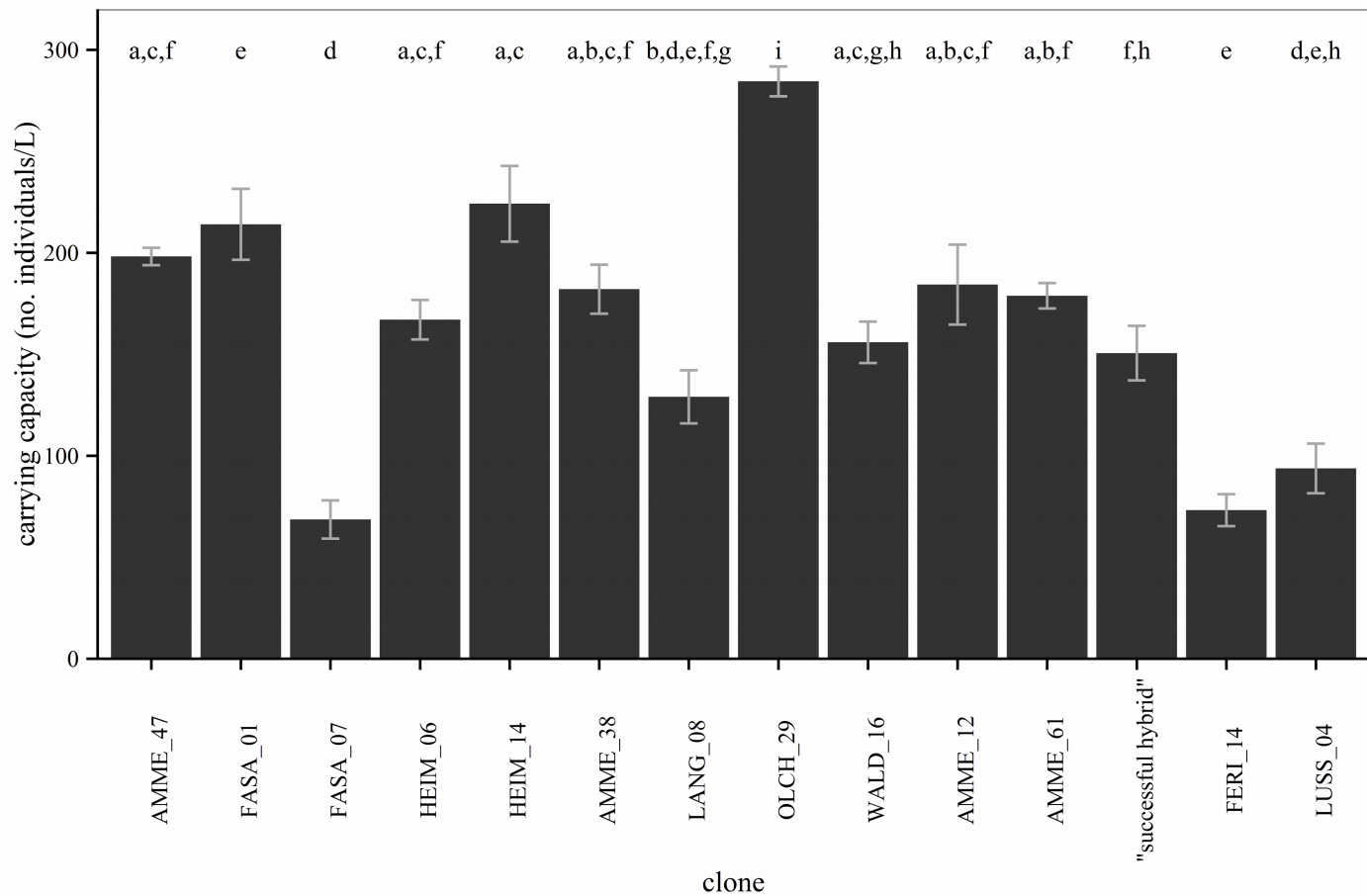

Supplement: S8 Fig — For taxon memberships see Table 1. Shown are the means ± S.E. Same letters above the columns indicate no significant differences between the respective clones. (PDF) [file pone.0140275.s008.pdf]

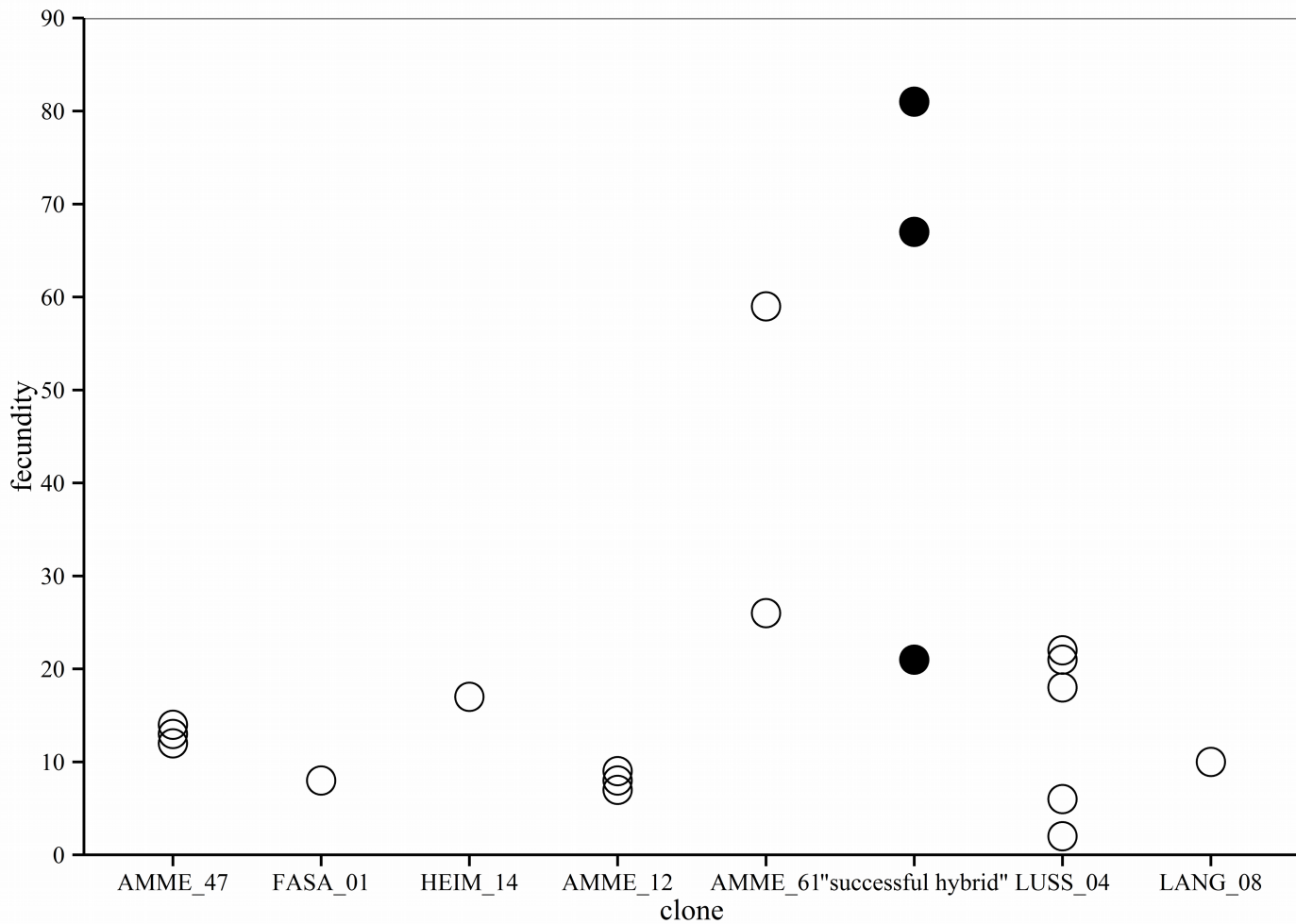

Supplement: S10 Fig — Values are provided per each individual replicate, but only for those individuals that have reproduced at least once. For taxon membership see Table 1. (PDF) [file pone.0140275.s010.pdf]
