# Supplementary material for: Extreme Environments Facilitate Hybrid Superiority – The Story of a Successful Daphnia galeata × longispina Hybrid Clone
Source: PLoS One. 2015 Oct 8;10(10):e0140275. doi: 10.1371/journal.pone.0140275 (PMC4598010; doi:10.1371/journal.pone.0140275)
Supplement: S9 Fig — In addition, number of clutches and fecundity versus time is shown for the Cox proportional hazard model fit. The solid lines (βt) give the estimated effect of the predictors through time in the experiment (with 95% confidence interval). (PDF) [file pone.0140275.s009.pdf]

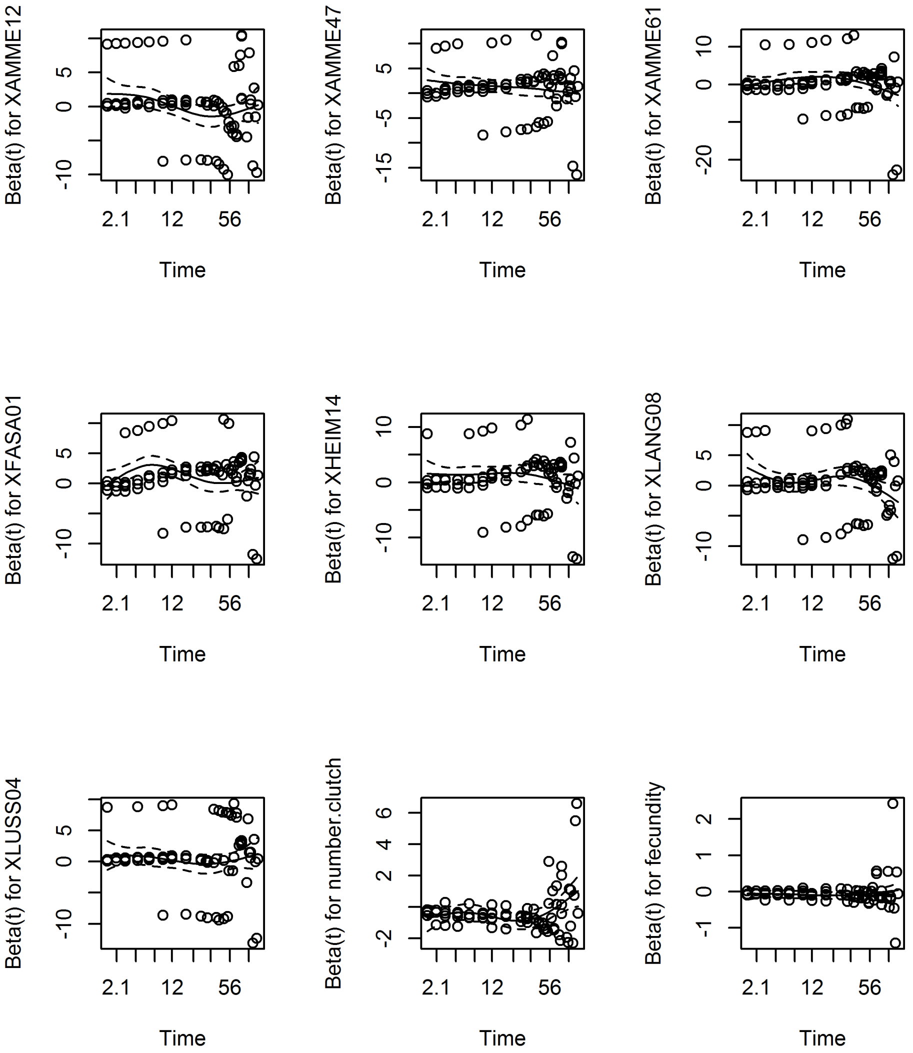
Beta(t) for XAMME12
Time
2.1
12
56
-10
0
5
Beta(t) for XAMME47
Time
2.1
12
56
-15
-5
5
Beta(t) for XAMME61
Time
2.1
12
56
-20
0
10
Beta(t) for XFASA01
Time
2.1
12
56
-10
0
5
Beta(t) for XHEIM14
Time
2.1
12
56
-10
0
10
Beta(t) for XLANG08
Time
2.1
12
56
-10
0
5
Beta(t) for XLUSS04
Time
2.1
12
56
-10
0
5
Beta(t) for number.clutch
Time
2.1
12
56
-2
2
4
6
Beta(t) for fecundity
Time
2.1
12
56
-1
0
1
2
