# Supplementary material for: Extreme Environments Facilitate Hybrid Superiority – The Story of a Successful Daphnia galeata × longispina Hybrid Clone
Source: PLoS One. 2015 Oct 8;10(10):e0140275. doi: 10.1371/journal.pone.0140275 (PMC4598010; doi:10.1371/journal.pone.0140275)
Supplement: S1 Table — (PDF) [file pone.0140275.s011.pdf]

experiment	number of clones	no. of replicates	experimental unit	mg C L^{-1} *S. obliquus*	feeding frequency	light/dark photoperiod	media exchange	termination of the experiment	parameters measured	measurement frequency
competition	38 *Daphnia*<br>2 *Simocephalus*	4	multiple individuals	1	every third day	12:12	no exchange	ten weeks	population density,<br>final frequency of genotypes	once in the end
temperature	29 *Daphnia*	6 to 10	single individual	1	daily	12:12	every third day	after the 3rd clutch	age at first clutch release,<br>number of offspring in the first clutch,<br>total number of offspring in the first three clutches,<br>first clutch offspring body length,<br>body length of experimental mothers	daily
crowded	6 *Daphnia*	10	single individual	1	daily	12:12	daily	after the 3rd clutch	age at first clutch release,<br>number of offspring in the first clutch,<br>total number of offspring in the first three clutches,<br>first clutch offspring body length,<br>body length of experimental mothers	daily
carrying capacity	14 *Daphnia*	6	multiple individuals	1	every third day	12:12	weekly	seven weeks	population size	weekly
overwintering	13 *Daphnia*	15	single individual	0.1	every third day	08:16	weekly	untill the last experimental individual died	lifespan,<br>total number of offspring from all clutches,<br>total number of clutches,<br>number of ephippia produced	every second day
