# Supplementary material for: Extreme Environments Facilitate Hybrid Superiority – The Story of a Successful Daphnia galeata × longispina Hybrid Clone
Source: PLoS One. 2015 Oct 8;10(10):e0140275. doi: 10.1371/journal.pone.0140275 (PMC4598010; doi:10.1371/journal.pone.0140275)
Supplement: S2 Table — (PDF) [file pone.0140275.s012.pdf]

		clonal set				
clonal ID	taxon	1	2	3	4	5
AMME_10	*D. galeata*			×		
AMME_24	*D. galeata*		×			
AMME_47	*D. galeata*	×				
AMME_58	*D. galeata*					×
AMME_66	*D. galeata*				×	
FASA_01	*D. galeata*	×		×		
FASA_07	*D. galeata*		×		×	
FASA_13	*D. galeata*					×
HEIM_05	*D. galeata*				×	
HEIM_06	*D. galeata*		×			
HEIM_08	*D. galeata*					×
HEIM_12	*D. galeata*			×		
HEIM_14	*D. galeata*	×			×	
LERC_09	*D. galeata*					×
LERC_11	*D. galeata*		×	×	×	
LERC_33	*D. galeata*	×				
AMME_38	*D. longispina*		×	×		
LANG_08	*D. longispina*		×			×
LANG_21	*D. longispina*				×	
LANG_26	*D. longispina*	×				
LUSS_12	*D. longispina*			×		×
LUSS_30	*D. longispina*	×			×	
OLCH_02	*D. longispina*				×	
OLCH_17	*D. longispina*					×
OLCH_22	*D. longispina*	×		×		
OLCH_29	*D. longispina*		×			
WALD_03	*D. longispina*	×				
WALD_05	*D. longispina*			×		
WALD_12	*D. longispina*				×	
WALD_16	*D. longispina*		×			
WALD_37	*D. longispina*					×
AMME_03	F1-hybrid				×	×
AMME_12	F1-hybrid			×		
AMME_61	F1-hybrid	×	×			
„successful hybrid“	F1-hybrid	×	×	×	×	×
FERI_01	F1-hybrid	×				
FERI_14	F1-hybrid		×		×	
LUSS_04	F1-hybrid			×		×
BOHM_01	*Simocephalus*		×			×
BOHM_03	*Simocephalus*	×		×	×	
