# Supplementary material for: Extreme Environments Facilitate Hybrid Superiority – The Story of a Successful Daphnia galeata × longispina Hybrid Clone
Source: PLoS One. 2015 Oct 8;10(10):e0140275. doi: 10.1371/journal.pone.0140275 (PMC4598010; doi:10.1371/journal.pone.0140275)
Supplement: S3 Table — The direction of a difference is indicated by < / > signs. Significant p-values after Bonferroni-Holm correction are shown in bold. (PDF) [file pone.0140275.s013.pdf]

experiment		temperature					crowded				
		age at 1st clutch release	no. of offspring in the 1st clutch	total no. of offspring in the 1st three clutches	1st clutch offspring body length	body length experimental mothers	age at 1st clutch release	no. of offspring in the 1st clutch	total no. of offspring in the 1st three clutches	1st clutch offspring body length	body length experimental mothers
AMME_10	*D. galeata*										
AMME_24	*D. galeata*	15°C>20°C, p=0.009	-	-	-	-					
AMME_47	*D. galeata*	15°C>20°C, p=0.004	-	-	-	-					
FASA_01	*D. galeata*	15°C>20°C, p=0.005	-	-	-	-	-	C>NC, p=0.03	-	C>NC, p=0.02	C>NC, **p=0.002**
FASA_07	*D. galeata*	15°C>20°C, p=0.005	-	-	-	-					
FASA_13	*D. galeata*	-	-	-	-	-					
HEIM_06	*D. galeata*						-	C>NC, p=0.03	C>NC, **p=0.01**	C>NC, **p=0.001**	C>NC, **p=0.001**
HEIM_08	*D. galeata*	-	-	-	-	-					
HEIM_14	*D. galeata*	15°C>20°C, p=0.01	-	-	-	-					
LERC_09	*D. galeata*										
LERC_11	*D. galeata*	15°C>20°C, **p=0.001**	-	-	15°C<20°C, p=0.02	15°C<20°C, p=0.01					
LERC_33	*D. galeata*	15°C>20°C, p=0.004	-	-	-	-					
AMME_38	*D. longispina*	15°C>20°C, p=0.007	-	-	-	-	-	-	-	-	C>NC, **p=0.05**
LANG_08	*D. longispina*	-	-	-	15°C<20°C, p=0.004	-					
LANG_21	*D. longispina*	15°C>20°C, p=0.03	-	15°C<20°C, p=0.04	15°C<20°C, p=0.03	-					
LANG_26	*D. longispina*	15°C>20°C, p=0.01	-	-	15°C<20°C, p=0.01	-					
LUSS_12	*D. longispina*										
LUSS_30	*D. longispina*	-	-	-	-	-					
OLCH_02	*D. longispina*	15°C>20°C, p=0.003	-	-	-	-					
OLCH_17	*D. longispina*										
OLCH_29	*D. longispina*	15°C>20°C, p=0.02	-	-	-	-	-	C>NC, p=0.01	**C>NC, p<0.001**	**C>NC, p<0.001**	**C>NC, p=0.006**
WALD_03	*D. longispina*	-	-	-	15°C<20°C, p=0.008	15°C<20°C, p=0.02					
WALD_05	*D. longispina*										
WALD_16	*D. longispina*	-	-	-	15°C<20°C, p=0.004	-					
AMME_03	F1-hybrid	15°C>20°C, **p=0.002**	-	-	-	-					
AMME_12	F1-hybrid						-	-	-	-	C>NC, **p=0.01**
AMME_61	F1-hybrid	15°C>20°C, p=0.006	-	-	-	15°C<20°C, p=0.03					
"successful hybrid"	F1-hybrid	15°C>20°C, **p<0.001**	-	-	15°C<20°C, p=0.02	-	C<NC, **p=0.004**	-	C>NC, p=0.03	-	C>NC, **p<0.001**
FERI_01	F1-hybrid	15°C>20°C, p=0.01	-	-	-	-					
FERI_14	F1-hybrid										
LUSS_04	F1-hybrid	15°C>20°C, p=0.007	-	-	-	-					
